# Reducing mapping reference and lineage bias in Mycobacterium tuberculosis

**DOI:** 10.1099/mgen.0.001690

**Published:** 2026-04-10

**Authors:** Arturo Torres Ortiz, Xavier Didelot, Louis Grandjean

**Affiliations:** 1 Johns Hopkins Bloomberg School of Public Health, Baltimore, MD 21205, USA; 2 Department of Infection, Immunity and Inflammation, Institute of Child Health, UCL, London WC1N 1EH, UK; 3 School of Life Sciences and Department of Statistics, University of Warwick, Coventry, CV4 7AL, UK

**Keywords:** bioinformatics, genome assembly, mapping, next-generation sequencing, tuberculosis

## Abstract

Whole-genome sequencing provides a vast amount of genetic information, but its use in clinical and epidemiological studies often depends on the accurate inference of genomic variants. Comparative genomic studies in *Mycobacterium tuberculosis* typically involve mapping short reads from a diverse population to the same reference genome. This approach can lead to the incorrect characterization of many genomic regions that are susceptible to mapping bias when the reference is too distantly related to the sample. We analysed the consequences of mapping reads from different lineages of *M. tuberculosis* to the commonly used reference H37Rv and showed that the mapping bias varied depending on both the lineage and the gene mapped. To resolve these issues, we propose a new hybrid workflow which involves three steps: first, building a *de novo* assembly from short reads; second, aligning this assembly to a reference genome; and finally, mapping the reads to this aligned assembly. We show that many of the lineage and gene biases were corrected using this approach, which leads to a better characterization of lineages and hypervariable regions in comparative analysis. Our proposed approach will enable researchers to elucidate more genetic variations in *M. tuberculosis* and other bacterial pathogens.

**Impact Statement**

Reference genomes are essential for many genomic applications in *Mycobacterium tuberculosis* research. Common short-read approaches involve mapping sequencing reads to the reference genome, typically H37Rv. However, the choice of reference can affect the results, especially when the genetic distance between the reference and the sample is high. We propose a bioinformatic workflow that reduces reference short-read mapping bias by including an initial assembly step used to create a sample-specific reference genome, minimizing the distance between target and query sequences during mapping. We leveraged data from public repositories and simulations of sequencing reads to show an increased accumulation of variant calling errors when using the common workflow of read mapping compared to our proposed assembly and mapping approach. Reference bias was most pronounced in lineage 2 and in genes containing low complexity and repetitive regions, such as the *pe/ppe* gene family, insertion sequences and genes involved in cell wall processes. In all lineages and gene categories, our suggested workflow reduced the bias produced by the choice of reference genome, leading to more accurate results.

## Data Summary

All raw sequencing data used for this study are publicly available. The accession numbers are listed in Supplementary Material 1, available in the online Supplementary Material. All custom code used in this article can be accessed at https://github.com/arturotorreso/tb_refBias.git.

## Introduction

Whole-genome sequencing has become a critically important tool in pathogen microbiology [1]. The analysis of genomic data in *Mycobacterium tuberculosis* is currently standard in many clinical and public health settings. Over the last decade, researchers have identified drug-resistant-associated variants that are used alongside culture methods for drug susceptibility testing [2] and, in some settings, have completely replaced them for the detection of resistance to first-line drugs [3]. Moreover, genomic sequences are now used in place of spoligotyping for genotyping studies, and they are increasingly used for epidemiological surveillance and contact investigation [4]. As whole-genome sequencing is increasingly used to influence patient outcomes and public health practices, the robustness of the results becomes more crucial.

Most bioinformatic workflows used in *M. tuberculosis* are based on resequencing approaches, which consist of genome assembly guided by mapping of short reads against a reference genome. This strategy is favoured in *M. tuberculosis* due to the existence of a well-annotated reference genome since 1998 based on the H37Rv strain [5], a low mutation rate and a high clonality characterized by the absence of homologous recombination and little variation in accessory genomic content [6]. However, some regions present higher variability than others [7], which may hinder correct mapping of the short reads to the reference genome. Moreover, duplicated regions also bias short-read mapping, as reads map to several locations with similar mapping quality. As a consequence, most bioinformatic workflows in *M. tuberculosis* remove problematic regions such as the *pe* and *ppe* gene families [8]. The mapping bias is exacerbated by the fact that all lineages of *M. tuberculosis* are compared to the same H37Rv reference, which belongs to lineage 4.9. Therefore, strains highly divergent from the reference genome are expected to be less accurately characterized.

Using a common reference genome is nevertheless often necessary in *M. tuberculosis* research, as many lineages do not have well-annotated references. Moreover, whole-genome comparative projects require all sequences to share the same genomic coordinates so that polymorphisms become evident. For example, genome-wide association studies (GWAS) compare nucleotides at each polymorphic position in order to link genetic variation to phenotypes of interest, such as drug resistance or pathogenicity [9]. Similarly, having all samples within the same genomic coordinates is a prerequisite for phylogenetic inference, as it considers the relatedness of genetic sequences in terms of evolution at each site [10]. Given that most genomic pipelines currently analyse whole-genome sequences of hundreds and thousands of samples, alignment of all the sequences is computationally intensive and harder to parallelize, and it is not realistic for aligning complete genomes. Therefore, most projects involve mapping of short reads to a complete genome and representing sequences on the same coordinates.

In this study, we investigate how the genetic diversity in *M. tuberculosis* affects the reconstruction of genome sequences using short reads and characterize regions of high mapping errors in the *M. tuberculosis* genome by using simulations of short reads from completely assembled genomes. Additionally, we propose a new hybrid approach using *de novo* assembly and mapping of short reads to a sample-specific reference genome. We show that this reduces the number of errors in many of the problematic regions. The patterns seen in simulations were also confirmed using real short reads. The proposed method first performs *de novo* assembly of short reads into contigs, which are, in turn, aligned to the H37Rv reference genome, taking advantage of the fact that longer sequences have an increased probability of mapping uniquely and with good quality to the target sequence. From the aligned contigs, a sample-specific reference genome is inferred, against which the short reads are subsequently mapped. We then demonstrate that using a reference tailored to the sample will allow for a better characterization of the *M. tuberculosis* genome and reduce mapping bias from short-read sequencing technologies.

## Methods

### Genome assembly collection

Complete *M. tuberculosis* genome assemblies were downloaded from the NCBI Assembly database [11]. To ensure high-quality references, assemblies constructed exclusively using short-read sequencing were excluded. Assemblies were kept for further analysis if the sequencing technology used for sequencing was at least one of PacBio (Pacific Biosciences), Sanger sequencing or a hybrid combination of Nanopore and Illumina. Assembly quality was verified for all genomes using CheckM v1.2.5 (genus *Mycobacterium* taxonomy workflow) and BUSCO v6.0.0 with the universal bacteria_odb10 lineage dataset. Lineages and sublineages were inferred from the assemblies by mapping them to the H37Rv reference genome (GenBank accession code AL123456) with minimap2 v2.28 [12] and using lineage-specific SNPs [13]. Only assemblies from *M. tuberculosis* lineages 1, 2, 3 and 4 were kept for further analysis. Assembly information and quality metrics for each assembly are provided in Supplementary Material 1.

### Whole-genome sequence analysis

**Fig. 1.**
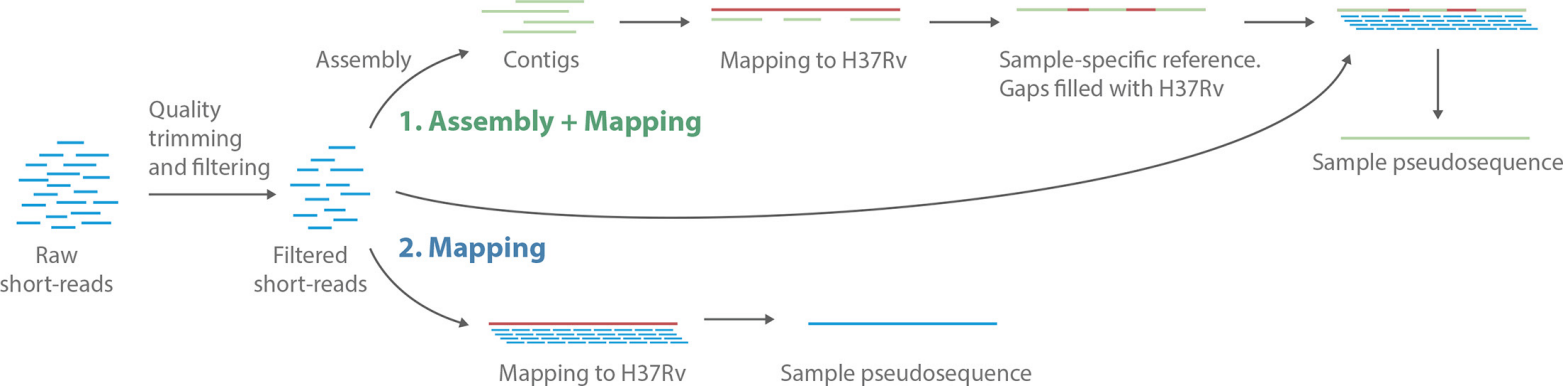
Overview of the two pipelines compared in this study. Two different pipelines using Illumina short reads were compared. First, a novel hybrid assembly and mapping workflow, where short reads are *de novo* assembled and the resulting contigs are mapped against the reference genome H37Rv. Unmapped regions and gaps are filled using the H37Rv reference to construct a sample-specific reference genome against which short reads are mapped. To prevent coordinate shifts, insertions are excluded from the sample-specific reference genome. Second, a common workflow of short-read mapping against the H37Rv reference genome.

For each assembly, ten sets of short-read sequencing raw reads were simulated using ART v2.5.8 [14] with the built-in quality and error profile for HiSeqX TruSeq Illumina sequencing systems. Mean read length of the simulated reads was set to 150 bp, mean coverage of 50× and mean and sd for insert sizes between pairs of 650 and 150 bp, respectively. The resulting reads were analysed using two pipelines (Fig. 1): a standard mapping approach and a hybrid three-step workflow involving *de novo* assembly, alignment and mapping.

For the mapping pipeline, short raw reads were trimmed for low-quality bases using Trimmomatic v0.39 [15] with a minimum mean quality per base of 20 in a 4-base-wide sliding window. The five leading and trailing bases of each read were removed, and reads with an average quality lower than 20 were discarded. The resulting reads were mapped to the H37Rv reference genome (GenBank: AL123456) using BWA v0.7.17 [16] with the mem algorithm. The reads were processed using SAMtools v1.10 [17], and duplicates were marked using Sambamba v0.8.0 [18]. Genetic variants in reference to H37Rv were called using BCFtools v1.10.2 [19], and variants were soft-filtered with a minimum mapping quality of 20, a base quality of 20, a minimum depth of 20 and a minimum allele depth of 10 with 3 reads in both the forward and reverse strands, as well as position bias, mapping quality bias and strand bias. Marked duplicates are ignored by default when calling variants using BCFtools.

The proposed hybrid approach was performed as follows. First, the short reads were quality-filtered using Trimmomatic v0.39 as described above. *De novo* assembly of the short reads was performed using the SPAdes genome assembler v3.14.0 [20] using kmer sizes of 21, 33, 55, 77 and 101 bp. The resulting contigs were filtered by removing contigs that are assembled with less than two reads and that have a length of 300 bp. The filtered contigs were mapped to the H37Rv reference genome using minimap2 [12] with the asm20 preset. While *M. tuberculosis* generally shows low genome-wide diversity, the relaxed alignment penalties of the asm20 preset were chosen to ensure alignment across hypervariable regions and divergent lineages. Variants were identified from the contig alignments using BCFtools as described above. A sample-specific consensus sequence was then generated by incorporating these variants into the H37Rv sequence using the vcf2pseudoseq.py script. To prevent coordinate shifts and maintain alignment with the H37Rv coordinate system, insertions were excluded from the sample-specific reference genome. Any unmapped regions, deletions and ambiguous bases were filled with the H37Rv reference sequence to maintain a standardized coordinate system for downstream comparative analysis. The inferred sequence was used as a sample-specific reference genome, against which the filtered short reads were mapped using BWA and Sambamba, and variants were called using BCFtools, as previously described.

### Calculation of error rates and distribution

The high-quality complete genome assemblies downloaded from the NCBI database were aligned to the H37Rv genome using minimap2 to be used as the ground truth for comparison with the variant calls produced by the two short-read workflows. The VCF files were compared to their respective initial complete assembly using the VCFR package [21] within the R statistical software. Structural variants, insertions and deletions were not considered for the comparison. All genomic positions within each sample in which either the complete assembly or the sample VCF showed an alternate allele were compared. A false positive was defined when the sample showed an alternate allele, whereas the complete assembly did not. A false negative was defined as the presence of a reference allele in the sample, while the complete assembly showed an alternate allele when compared to H37Rv. The error rate for each gene was defined as the number of false positives plus the number of false negatives, divided by the length of the gene. The error rate was calculated for each gene within each simulated read set.

To model the error distribution per gene and lineage, the error rate of all samples and replicates was compared to a null distribution, which was estimated by calculating the error rate in 100 random genomic intervals of length equal to the specific gene. The fitted error rate and CIs for each lineage were calculated by comparing the error rate to that of the null distribution in a linear model.

### Phylogenetic analysis

Phylogenetic trees were inferred using RAxML-NG v1.2.1 [22] with a GTR+*ω*, 20 starting trees (10 random and 10 parsimony), a minimum branch length of 10−9 and Stamatakis ascertainment bias correction to account for the absence of monomorphic sites in the alignment. Downstream phylogenetic analyses were performed using the R package ape [23].

## Results

### Genome selection

A dataset of 336 *M*. *tuberculosis* complete genomes from 96 different projects was collected from the NCBI Assembly database (Supplementary Material 1) . The majority of these complete genomes were sequenced with third-generation sequencing methods, especially with the PacBio platform (123 out of 336) and Oxford Nanopore (18 out of 336). A total of 111 genomes were assembled using only Illumina short-read platforms. A combination of short and long reads was used in 63 assemblies. The remaining isolates were sequenced with other technologies.

Using lineage-defining SNPs [13], the majority of the assemblies were classified as *M. tuberculosis* lineage 4 (50%, 167 out of 336), while 8% were lineage 1 (28 out of 336), 27% were lineage 2 (91 out of 336) and 4% were lineage 3 (13 out of 336). The remaining assemblies were assigned to minor lineages (4%, 12 out of 336), as well as animal-adapted lineages such as *Mycobacterium bovis* and *Mycobacterium microti* (7%, 25 out of 336).

After filtering for high-quality sequencing platforms (see Methods), 183 assemblies remained. A subset of 159 assemblies belonged to lineages 1 to 4 and thus were kept for analysis. Of the 159 assemblies, 22 were classified as lineage 1, 73 as lineage 2, 10 as lineage 3 and 54 as lineage 4. We validated the assembly quality of the 159 selected assemblies. All assemblies were complete, consisting of a single contig with 0% gaps. The median CheckM and BUSCO completeness scores across the dataset were 98.4%. Median CheckM contamination was 0.37% (Supplementary Material 1).

### Mapping reference bias in *M. tuberculosis*

To understand the intrinsic bias of mapping short reads to the H37Rv reference genome, we simulated Illumina short reads and mapped them to the H37Rv reference genome using two different workflows (Fig. 1). In the first workflow, we repeated a frequently used mapping approach in which short reads are directly mapped against the H37Rv reference genome. In contrast, our proposed hybrid workflow extends on this method by adding preliminary steps in which *de novo* assembly is used to assemble the short reads into longer contigs which are then mapped against the H37Rv reference genome. Unmapped regions are filled with reference sequences to construct a sample-specific reference against which the short reads can be mapped (Fig. 1).

**Fig. 2.**
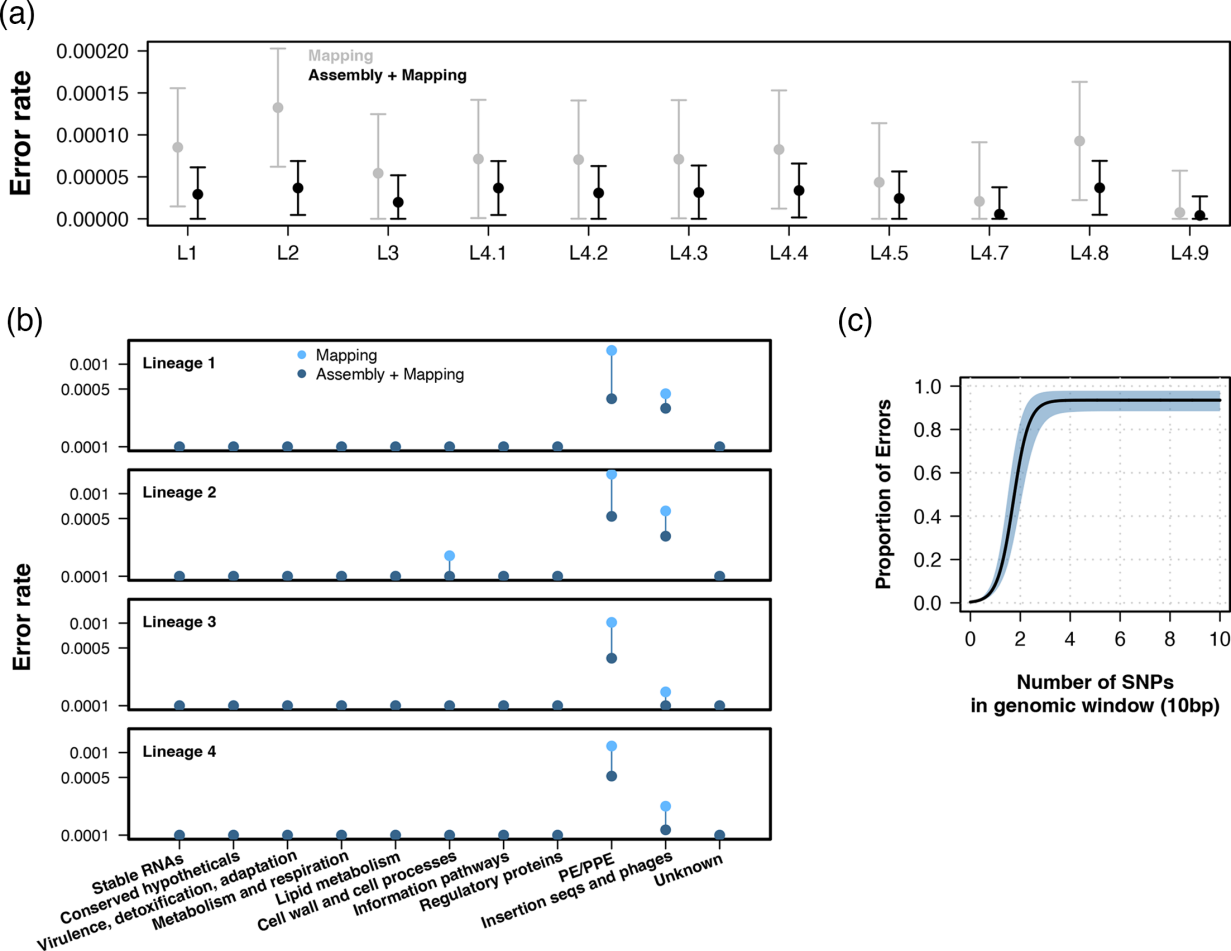
Variant calling error depends on *M. tuberculosis* lineage and genomic structure. (a) Error rate by lineage estimated from a linear model. Error rate is specified in the y-axis, while each lineage is shown in the x-axis. Best estimates are shown as dots. Error bars represent the 95% CI. Grey shows the error rate using a standard mapping approach, while black shows the error profiles using our proposed hybrid pipeline. (b) Error rate (y-axis) by gene functional category (x-axis) and lineage. Light and dark blue show the error rate for the mapping approach and the hybrid pipeline, respectively. (c) Distribution of variant calling errors as a proportion of the total number of bases (y-axis) in genomic windows of 10 bp (x-axis). The solid line shows the best fit, while the shaded blue area represents the 95% CI.

By comparing the distribution of errors per gene against a null distribution calculated using genomic windows of different sizes, we inferred the error rate per base for each gene and lineage in a linear model. Lineage 2 had the highest error rate (1.3×10^−4^, 6.2×10^−5^ – 20.0×10^−4^ 95% CI), followed by lineage 4.8, lineage 4.4 and lineage 1 (all *P*-values<0.05, Fig. 2a). Independent of lineage, higher error rates were estimated for *pe*/*ppe* gene families, as well as insertion sequences and phages (Fig. 2b). Genes functionally related to cell wall processes had a higher error rate exclusively for lineage 2 samples (Fig. 2b). Areas of the genome with a high density of SNPs were correlated with a higher proportion of variant calling errors. Using a genomic window of 10 bp and fitting a logistic model, we inferred that the number of errors rapidly increased as SNPs accumulate (Fig. 2c).

### Hybrid assembly of short reads decreases variant calling errors

The error rate and the CI of the error distribution using our proposed hybrid approach decreased for all lineages to a rate that was more uniform among them (Fig. 2a). Although error rates among *pe*/*ppe* and insertion sequences were still high when using the hybrid approach, the error rate for cell wall-related genes was reduced for lineage 2 to a level consistent with all other functional categories (Fig. 2b). To further quantify variant calling accuracy, we calculated genome-wide precision, recall and F1 scores per sample (Table 1). The mapping workflow resulted in a high median precision of almost 0.99 (IQR: 0.986–0.991) but a low median recall of 0.63 (IQR: 0.573–0.709), indicating a high rate of false negatives. The hybrid pipeline showed a median recall of 0.897 (IQR: 0.832–0.929) and a median precision of 0.983 (IQR: 0.947–0.99). The hybrid approach also showed a high median F1 score of 0.926 (IQR: 0.893–0.953), compared to 0.77 (IQR: 0.726–0.825) for the mapping pipeline.

**Table 1.** Performance metrics across pipelines

	Mapping	Assembly+mapping
Precision	0.989 (0.986–0.991)	0.983 (0.947–0.990)
Recall	0.630 (0.573–0.709)	0.897 (0.832–0.929)
F1	0.770 (0.726–0.825)	0.926 (0.893–0.953)

**Fig. 3.**
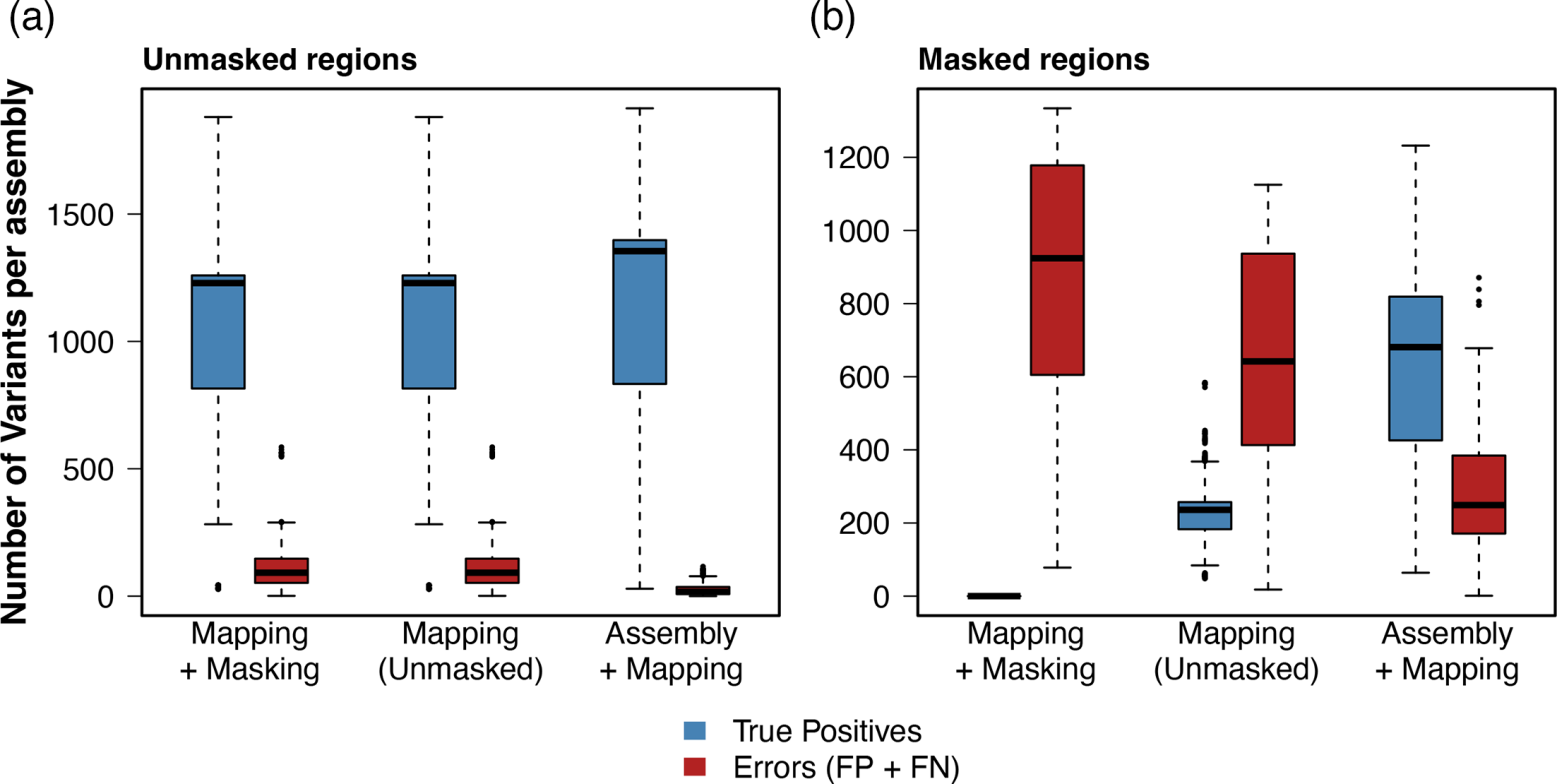
Comparison of variant calling performance across standard mapping, masking and hybrid pipelines. Boxplots representing the number of true-positive variants (blue) and errors (red, sum of false positives and false negatives) are shown per assembly across 1,590 simulated runs (159 assemblies with 10 replicates each). Data are separated into (a) conserved genomic regions and (b) hypervariable regions typically masked in tuberculosis genomic analysis. Masking approaches discard all true variants within the masked regions, resulting in false-negative errors.

We evaluated the performance of our hybrid approach against common practices that rely on masking hypervariable regions in the *M. tuberculosis* genome. We compared the number of true positives and errors (false positives and false negatives) per sample (Fig. 3). In unmasked genomic regions, the conventional mapping approach yielded a median of 1,229 true-positive variants (IQR: 815–1,259) and 92 errors (IQR: 52–147) per sample. The hybrid pipeline improved variant resolution in these conserved regions, recovering a median of 1,354.5 true positives (IQR: 833–1,397) and reducing the median errors to 17 (IQR: 8–36). In masked genomic regions, such as *pe/ppe* and insertion sequences, the masking filter removed a median of 924 true variants (IQR: 605–1,178) per sample, driven entirely by false negatives. Leaving these regions unmasked in the mapping-only approach recovered a median of 236 true variants (IQR: 183–257) but introduced 642 errors (IQR: 413–934) per assembly. By contrast, the hybrid pipeline identified 681 true variants (IQR: 426–819) while reducing the number of errors to 249 (IQR: 171–384).

**Fig. 4.**
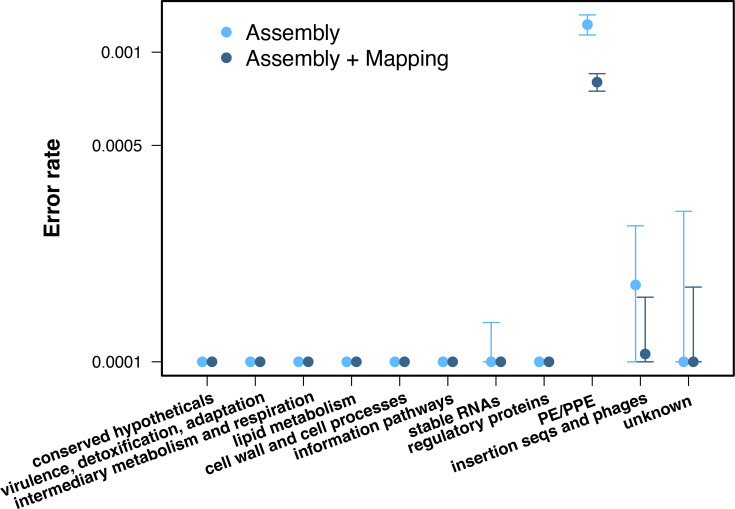
Error rate by gene functional category using real Illumina data. The y-axis represents the error rate per gene, calculated as the number of false-positive and false-negative variants divided by the length of the gene. Genes were grouped by gene categories (x-axis). Light and dark blue show the error rate for the conventional mapping approach and the recommended hybrid pipeline, respectively. CIs represent the 95% CI.

To test whether the results obtained with read simulations are consistent when using real data, we selected those complete assemblies that also had Illumina reads. Of the 159 assemblies analysed, only 20 had readily available Illumina short reads in public repositories. Overall, the patterns were similar to the ones described above on simulated data, with the highest error rates found in *pe/ppe* family genes and insertion and phage sequences (Fig. 4). For *pe/ppe* genes, the estimated difference error rate for the proposed hybrid pipeline was 8×10^−3^ (7.3×10^−3^–8.7×10^−3^ 95% CI), almost one order of magnitude lower than with the conventional mapping workflow (1.2×10^−4^, 1.1×10^−4^–1.3×10^−4^ 95% CI).

**Fig. 5.**
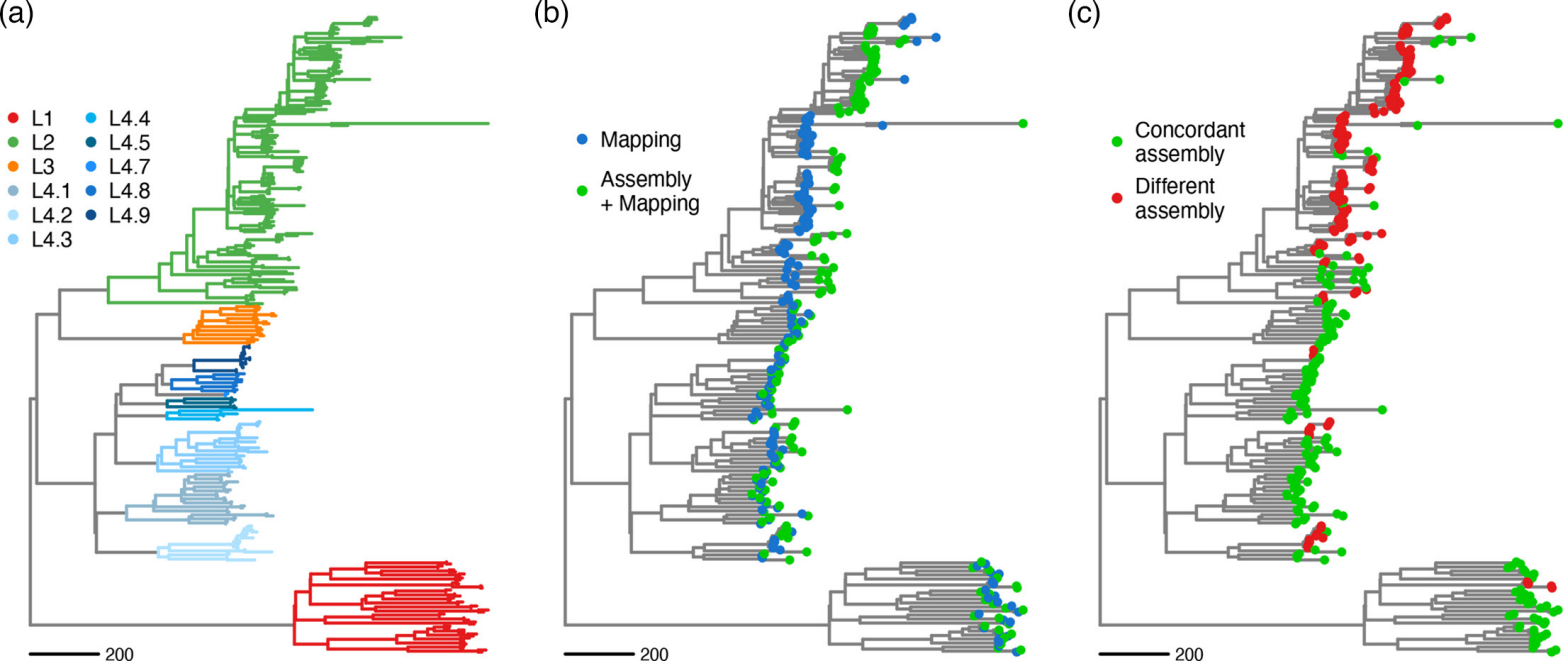
Phylogenetic inference of sequences derived from the mapping workflow and the hybrid approach. Phylogenetic tree of 163 isolates, each of them with two sequences constructed with the mapping pipeline and the hybrid approach. Genetic distance is depicted in substitutions per genome. When there is little difference between the pipelines for an isolate, sequences derived from both methods are expected to cluster together. (a) Colours distinguish between *M. tuberculosis* lineages and sublineages. (b) Blue tips show sequences derived using the conventional mapping approach, while green tips represent those constructed with the hybrid approach. (c) Isolates clustering together between the different pipelines are shown in green, while isolates that do not are shown in red.

We measured the impact of the different pipelines on phylogenetic analysis by inferring maximum-likelihood phylogenies using genome sequences from each pipeline and combining all sequences from both workflows. Isolates clustered together by lineage and sublineage regardless of the workflow used to construct the alignments (Fig. 5a). Sequences inferred from the same assembly are expected to cluster together and therefore share the same node in the phylogeny. We thus compared sequences inferred using the mapping approach and those inferred with the hybrid pipeline. Only 57% (90 out of 159) of the pairs of sequences inferred by the two pipelines were found to cluster together in the phylogeny. The clustering bias was more pronounced in lineage 2, where the isolates tended to cluster by pipeline rather than by sequence pair (Fig. 5b). Only 21% (15 out of 73) of lineage 2 assemblies clustered together, while all samples from lineage 3 (10 out of 10) and 91% (21 out of 22) of lineage 1 clustered together. All isolates from sublineages 4.1, 4.4, 4.5, 4.7 and 4.8 were clustered together, while 44% (4 out of 9) of lineage 4.2, 69% (9 out of 13) of lineage 4.3 and 86% (6 out of 7) of lineage 4.9 were clustered together (Fig. 5c). The clustering observed in lineage 2 by pipeline rather than isolate highlights the reference bias when the sample is highly divergent from the reference genome.

The effect of the last mapping step in our hybrid pipeline, where raw reads are mapped against the sample-tailored reference genome, was assessed by comparing the results obtained with our pipeline with those where contigs are aligned against the H37Rv genome, with no additional mapping step (Fig. S1). Both pipelines resulted in a lower error rate for all gene categories when compared to the mapping-only approach. However, the assembly-only pipeline (without final read mapping) had a slightly higher error rate for the gene categories *pe/ppe* and insertion and phage sequences. We further analysed those positions where the assembly-only pipeline was incorrect and the full hybrid pipeline gave the correct result. A total of 93% of incorrect positions were not covered by a contig in the assembly-only pipeline, compared to 73% of positions not covered overall for any incorrect position.

## Discussion

The slow mutation rate and low genetic diversity of *M. tuberculosis* contrast with its wide range of functional and phenotypic diversity. Apart from drug resistance genetic variants, little is known about genetic determinants of many phenotypes of clinical and epidemiological interest, such as active disease, virulence or transmissibility. Given the limitations of short reads in hypervariable or paralogous regions, a high proportion of the *M. tuberculosis* genome is not well defined in many studies attempting to understand the genetic basis of pathogen phenotypes. Most genomic projects in *M. tuberculosis* involve the study of multiple lineages. For comparative purposes, short reads are mapped to the reference genome H37Rv irrespective of the lineage of the sample. Since H37Rv belongs to the sublineage 4.9 of *M. tuberculosis*, this choice of reference may affect mapping of short reads when the genome of the sample that is being compared differs substantially from the target reference. In our work, we report lineage-specific error rates when mapping short reads to the reference genome H37Rv and show that many of the mapping biases can be ameliorated when using a hybrid strategy involving *de novo* assembly, alignment to the reference and short-read mapping.

Our proposed pipeline improves upon mapping of short reads by starting with an assembly step (Fig. 1). The resulting contigs are then aligned using the H37Rv genome as reference, filling in any gaps with H37Rv sequences, from which a sequence of the isolate within the H37Rv coordinates is derived. Finally, this sequence composed of an assembly with gaps filled with H37Rv sequences is used as a sample-specific reference genome to map the short reads in order to correct the assembly, calculate quality metrics and detect within-host diversity. The sample-specific reference genome increases the relatedness of the query and target sequences during short-read mapping and improves the characterization of highly divergent areas of the genome while maintaining H37Rv coordinates to allow for comparative analysis of multiple isolates. A hybrid assembly and mapping approach has been proposed for the study of HIV genomes from short-read sequences [24]. Our work focuses on the mapping bias in *M. tuberculosis* and proposes a similar pipeline using the H37Rv genome, which is the best annotated assembly and the most widely used reference in tuberculosis research.

The choice of reference genome affects the mapping of short reads, especially as the distance between the query sample and the reference increases [8]. Using short-read simulations of well-annotated assemblies, we show that mapping lineage 2 strains results in a higher error rate when compared to lineage 4.9 (Fig. 2a), which is the lineage of the H37Rv reference genome and thus the closest lineage to the reference assembly across our dataset. Mapping of short reads achieved a high genome-wide precision of almost 0.99, but it was characterized by a low recall of 0.63 and a median F1 score of 0.77. By using our hybrid assembly and mapping pipeline, we were able to maintain a high precision (0.98), while increasing the median recall to 0.90 and the F1 score to 0.93. Unsurprisingly, many of these errors accumulate in the *pe*/*ppe* gene families (Fig. 2b). The *pe*/*ppe* gene families represent about 10% of the *M. tuberculosis* genome [5] and are characterized by gene duplications and repetitive sequences, which make short-read mapping particularly challenging [25]. Similarly, we describe high error rates in insertion sequences and phages. Interestingly, genes linked to cell wall processes and host interaction had a high error rate exclusively in lineage 2 when compared to other lineages, especially genes from the *lpp* and *esx* gene families. Many of these errors are explained by the hypervariability of these regions and the accumulation of variants when compared to H37Rv, which result in soft masking of these regions by the mapper. Mapping short reads to hypervariable regions or different lineages could be improved by changing the parameters and penalties of the mapper. But the optimal parameters may be different for each lineage, which could result in mapping irregularities and make samples less comparable. Using our suggested hybrid approach, lineage differences in error rates were reduced. Moreover, the error rate in cell wall and host interaction-related genes for lineage 2 was as low as for the remaining lineages. Our results show that mapping biases caused by hypervariable regions can be overcome using a sample-specific reference built using *de novo* assembly. Common bioinformatic workflows in *M. tuberculosis* often mask these problematic regions to prevent the accumulation of false-positive variant calls. However, our results demonstrate that this masking strategy results in a substantial loss of genomic information (Fig. 3). Masking hypervariable regions successfully decreases the number of false positives but removes hundreds of biologically true variants per sample. Our hybrid approach decreases the number of errors in hypervariable regions compared to the mapping pipeline without introducing artefacts into conserved regions of the genome. Even though the mapping bias for *pe*/*ppe* gene families and phage sequences was reduced with our approach, their error rate remained high. Longer reads are thus required to better characterize these regions.

Our findings align with recent benchmarking using short- and long-read sequencing of *M. tuberculosis* isolates [26], which showed that conventional short-read mapping to H37Rv achieves high precision at the expense of recall, particularly in repetitive regions. While Marin *et al*. [26] reduced variant calling errors by optimizing quality thresholds and using conservative masking, overly stringent filtering discards true variation and can affect epidemiological inferences [8]. In fact, Walter *et al.* [8] showed that high conservative thresholds can yield worse results than more lenient filtering. Moreover, as discussed above, filtering optimized for a specific lineage may not be ideal for others. Our hybrid approach improves the low recall found in mapping-only approaches without requiring conservative filtering.

Our focus on comparative genomic analysis meant that the sample-specific reference had to be in the same coordinates as H37Rv so that polymorphisms can be directly detected. In order to do that, we aligned the *de novo* assembly against the H37Rv assembly and used the resulting alignment as the ground truth. Even though the alignment is facilitated by the length of the query sequence, there may still be mapping errors that affect the comparative analysis. The last step in our proposed pipeline, where short reads are mapped against a sample-tailored reference genome, is useful to obtain position depth and base quality statistics. Moreover, our simulations suggest that there is a small decrease in the error rate in *pe/ppe* genes and insertion sequences when using the additional mapping step. This improvement was mostly caused in positions not covered by contigs. In our suggested pipeline, these gaps between contigs would be covered by H37Rv sequences when constructing the sample-specific reference genome, which, in turn, will improve the mapping of the short reads. Future work could use lineage-specific reference genomes to further decrease the error rate in areas of the genome where short-read mapping is challenging.

To understand if the patterns observed in simulations were consistent in real data, we repeated the pipeline on the raw reads used to build the assemblies. Surprisingly, only 20 out of 159 samples had the raw data used to build the assembly available in public repositories, hindering reproducibility and the reuse of datasets. Despite this low level of data availability, we were able to replicate some of the patterns observed in the simulations, even without separating the data by lineages due to insufficient samples (Fig. 4). Similar to the simulations, the proposed assembly and mapping pipeline performed better than the conventional mapping one, especially in the *pe/ppe* gene families. There was also a reduction in the error rate for the insertion sequences, but the difference was not significant due to the wide CIs caused by the low sample size and the variability between lineages.

Given that each dataset has different read lengths and read quality, using simulations allowed us to model the intrinsic error rate of genome mapping with a consistent read length and sequencing error rate distribution. However, other biases introduced during library amplification or sequencing, such as those in regions with high GC content, are not taken into account in our analysis, and therefore, error rates in real data may vary from our estimates.

Phylogenetic inference was affected by the reference genome bias. To study the effect of pipeline choice in the tree, we combined the alignments for each assembly and pipeline and inferred a phylogeny. If both pipelines were giving similar results, we would expect the sequences from the same assembly to cluster together. This is indeed the case for a big part of lineage 1, lineage 3 and lineage 4. However, lineage 2 isolates clustered largely by pipeline (Fig. 5), suggesting that the genetic resolution gained by using the assembly and mapping pipeline was enough to guide sample clustering. Given that lineage 2 tends to display lower genetic diversity when mapping short reads to the H37Rv genome, changes in the sequence inference workflow would have a greater impact on clustering and phylogenetic methods.

A previous study had found that the choice of reference genome did not affect phylogenetic and epidemiological results [27]. However, this study was exclusively concerned with the effects on clustering of an outbreak of lineage 4 with relatively low genetic diversity. As all isolates carry the same biases when using a specific reference genome, clustering may be consistent, so phylogenetic analyses are not affected. In our study, we focused on high-resolution comparative analysis in each position, which may be biassed when using different reference genomes even if the broad clustering of the isolates is not. Even though clustering in our study was sometimes affected by the choice of pipeline, the relationship of the isolates within each pipeline was consistent, which may explain why Lee and Behr [27] did not find differences in clustering when using different reference genomes. Our work is thus more consistent with recent publications that look at false-negative and false-positive calls in short-read simulations [8].

Although our hybrid assembly and mapping approach improves the mapping bias in tuberculosis, there are several limitations that will affect any method that relies on a unified reference coordinate system. Since the sample-specific reference follows H37Rv coordinates, our pipeline focuses exclusively on the core genome shared with the reference. While this is common for comparative genomic workflows that include large sample sizes, such as GWAS or phylogenetic analysis, it excludes the *M. tuberculosis* pangenome. Novel lineage-specific insertions, accessory genes and some large-scale structural variations such as inversions or translocations will not be properly characterized. Second, genomic variants in highly divergent lineages may still be underrepresented if the assembled contigs cannot be confidently aligned to the H37Rv reference genome. Furthermore, our analysis focused on the major human-adapted lineages (lineages 1–4) due to the lack of enough data for other lineages. While these lineages represent the majority of the global tuberculosis burden, the extent of reference bias and performance of our proposed hybrid pipeline would need to be evaluated in other minor lineages. Therefore, while our proposed approach is advantageous for high-resolution comparative genomics, studies specifically investigating whole-genome structural variation or accessory gene content may still require reference-free *de novo* assembly methodologies. Furthermore, while the detection of large-scale structural variants is beyond the scope of this comparative resequencing study, the improved mapping accuracy and reduction in alignment noise achieved by our hybrid approach could theoretically improve the performance of dedicated structural variant callers that rely on sequencing depth, split read and discordant pair mapping signatures. Future research is needed to address the effect of an assembly and mapping hybrid approach on structural variant calling.

In conclusion, our results highlight the inaccuracies of short-read mapping in some regions of the genome when using a single unique reference genome, and how our proposed approach decreases the error rate and reference bias. Improving variant calling in hypervariable regions offers multiple advantages. First, variants in hypervariable gene families (such as *pe/ppe* and *esx* genes) are implicated in virulence and host–pathogen interactions, and thus improving the genetic resolution in those regions can, in turn, improve phenotypic associations between those variants and phenotypes of clinical relevance. Moreover, recovering true genetic variation that would otherwise be masked in outbreak isolates provides the increased phylogenetic resolution necessary to reconstruct transmission chains. Finally, capturing accurate variation in regions with high mutation rates will improve phylogenetic estimates and molecular clock estimates. Therefore, decreasing the ambiguity in short-read sequences will allow researchers to include more regions for analysis, elucidating new phenotypic associations, aiding transmission studies and increasing our understanding of the evolutionary landscape of *M. tuberculosis* and other micro-organisms.

## Supplementary material

10.1099/mgen.0.001690 Fig. S1.

10.1099/mgen.0.001690 Supplementary Material 1.

## Abbreviations

GWAS: genome-wide association studies
PacBio: Pacific Biosciences
