# Supplementary material for: Reducing mapping reference and lineage bias in Mycobacterium tuberculosis
Source: Microb Genom. 2026 Apr 10;12(4):001690. doi: 10.1099/mgen.0.001690 (PMC13068375; doi:10.1099/mgen.0.001690)
Supplement: Fig. S1. [file mgen-12-01690-s001.pdf]

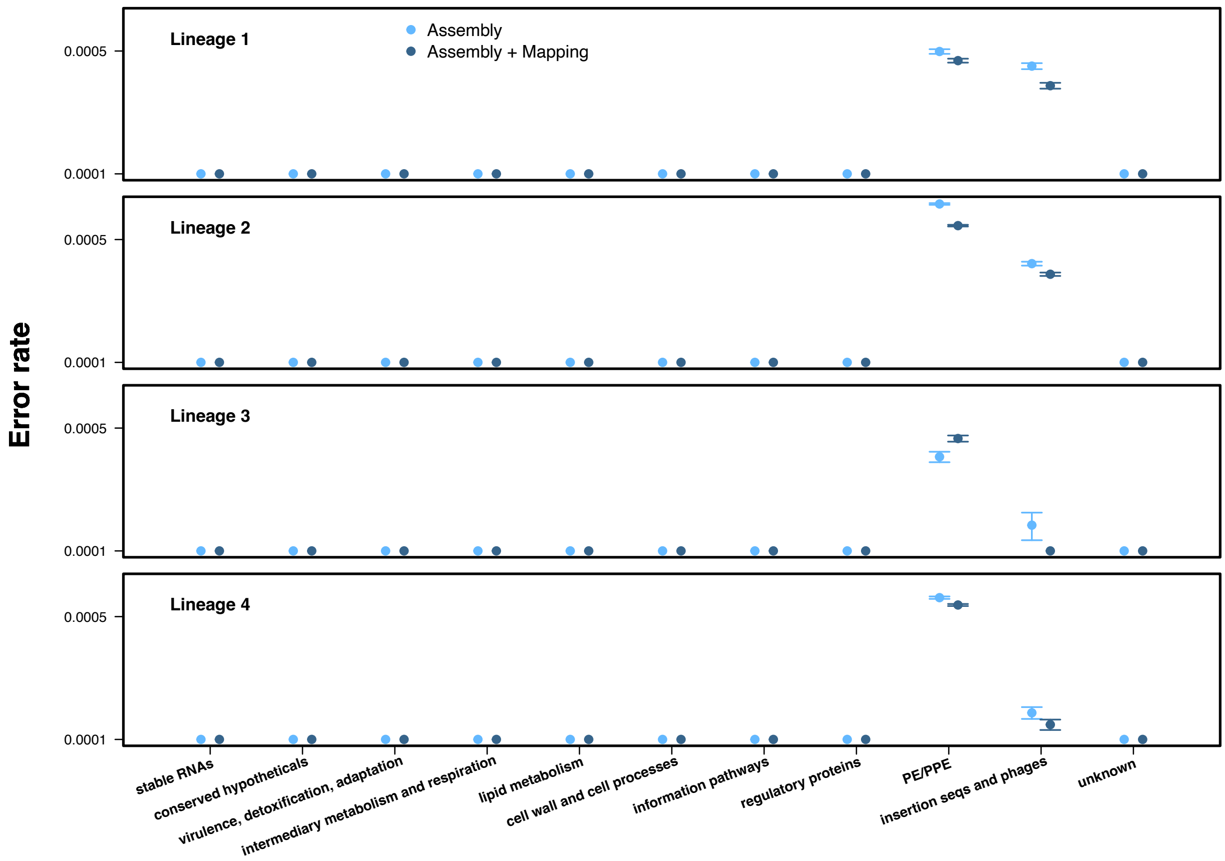

Supplementary Figure 1: Error rate for an assembly-only pipeline and the hybrid approach.
Error rate by gene functional category and lineage. Error rate was calculated as the number of false positives and false negative variants divided by the length of the gene. Genes were grouped in functional categories. Light and dark blue show the error rate for the mapping-only approach and the hybrid pipeline, respectively. Confidence intervals show the 95% CI.
