# Supplementary material for: Reducing mapping reference and lineage bias in Mycobacterium tuberculosis
Source: Microb Genom. 2026 Apr 10;12(4):001690. doi: 10.1099/mgen.0.001690 (PMC13068375; doi:10.1099/mgen.0.001690)
Supplement: Supplementary Material 1. [file mgen-12-01690-s002.pdf]

MyPDF

Assembly name	GenBank assembly accession	BioSample	BioProject	Date	Sequencing_technology	Lineage	Kept_analysis	Has_illumina_raw_reads	Number_of_Contigs	Total_Length_bp	Percent_Gaps	Contigs_N50	BUSCO_Completeness	CheckM_Completeness	CheckM_Contamination
ASM38990v1	GCA_000389905.1	SAMN02603650	PRJNA192916	5/8/13	illumina	Lineage 4.9	0	0	1	4404786	0.00%	4404780	63.7	90.35	0.33
ASM38992v1	GCA_000389925.1	SAMN02603652	PRJNA193386	5/8/13	illumina	Lineage 4.5	0	0	1	4392876	0.00%	4392876	66.9	89.66	0.67
ASM27708v1	GCA_000277085.1	SAMN02603736	PRJNA73717	3/20/12	roche_454	Lineage 4.3	0	0	1	4380119	0.00%	4380119	73.4	96.04	0.33
ASM27710v1	GCA_000277105.1	SAMN02603737	PRJNA73719	3/20/12	roche_454	Lineage 1.2	0	0	1	4406587	0.00%	4406587	79.8	95.5	0
ASM154473v1	GCA_001544735.1	SAMN03253093	PRJNA270004	2/2/16	pacbio	M. bovis	0	0	1	4351313	0.00%	4351313	83.9	95.37	1
ASM154485v1	GCA_001544855.1	SAMN03257091	PRJNA270004	2/2/16	pacbio	Lineage 6.1	0	0	1	4386422	0.00%	4386422	84.7	95.92	0
ASM154493v1	GCA_001544935.1	SAMN03257093	PRJNA270004	2/2/16	pacbio	Lineage 2.2	0	0	1	4405033	0.00%	4405033	89.5	96.75	0
ASM154481v1	GCA_001544815.1	SAMN03257090	PRJNA270004	2/2/16	pacbio	M. microti	0	0	1	4370115	0.00%	4370115	91.1	99.27	0
ASM27034v1	GCA_000270345.1	SAMN02603010	PRJNA19585	7/5/11	sanger;illumina	Lineage 2.2	1	0	1	4398812	0.00%	4398812	92.7	98.45	0
ASM154498v1	GCA_001544985.1	SAMN03257095	PRJNA270004	2/2/16	pacbio	Lineage 4.4	0	0	1	4417090	0.00%	4417090	93.5	98.89	0

Assembly name	GenBank assembly accession	BioSample	BioProject	Date	Sequencing_technology	Lineage	Kept_analysis	Has_illumina_raw_reads	Number_of_Contigs	Total_Length_bp	Percent_Gaps	Contigs_N50	BUSCO_Completeness	CheckM_Completeness	CheckM_Contamination
ASM154489v1	GCA_001544895.1	SAMN03257092	PRJNA270004	2/2/16	pacbio	Lineage 2.2	0	0	1	4419839	0.00%	4419839	94.4	97.7	0
ASM289518v2	GCA_002895185.2	SAMN07125453	PRJNA386696	3/19/18	illumina; nanopore	Lineage 2.2	1	0	1	4404064	0.00%	4404064	94.4	98.7	0
ASM1104445v1	GCA_011044455.1	SAMN14150054	PRJNA607885	3/1/20	illumina; nanopore	Lineage 2.2	1	0	1	4418311	0.00%	4418311	94.4	98.92	0
ASM154467v1	GCA_001544675.1	SAMN03253054	PRJNA270004	2/2/16	pacbio	Lineage 4.9	1	0	1	4428621	0.00%	4428621	95.2	98.86	0.67
ASM95415v1	GCA_000954155.1	SAMN03258416	PRJNA269967	3/17/15	pacbio	Lineage 2.2	1	0	1	4411216	0.00%	4411216	96	98.96	0
ASM27036v1	GCA_000270365.1	SAMN02603011	PRJNA19583	7/5/11	sanger;illumina	Lineage 2.2	1	0	1	4405981	0.00%	4405981	96.8	99.94	0
ASM70666v1	GCA_000706665.1	SAMN02767600	PRJNA246436	6/13/14	roche_454;illumina	Lineage 2.2	1	0	1	4410788	0.00%	4410788	96.8	99.94	0.33
ASM154501v1	GCA_001545015.1	SAMN03257096	PRJNA270004	2/2/16	pacbio	Lineage 4.2	1	0	1	4399422	0.00%	4399422	96.8	99.94	0
ASM154505v1	GCA_001545055.1	SAMN03257097	PRJNA270004	2/2/16	pacbio	Lineage 3	1	0	1	4426489	0.00%	4426489	96.8	99.67	0
ASM1489996v1	GCA_014899965.1	SAMN12325241	PRJNA555636	10/20/20	pacbio	Lineage 1.2	1	0	1	4447644	0.00%	4447644	96.8	99.61	0

Assembly name	GenBank assembly accession	BioSample	BioProject	Date	Sequencing_technology	Lineage	Kept_analysis	Has_illumina_raw_reads	Number_of_Contigs	Total_Length_bp	Percent_Gaps	Contigs_N50	BUSCO_Completeness	CheckM_Completeness	CheckM_Contamination
ASM2362v1	GCA_000023625.1	SAMN00763683	PRJNA21055	7/9/09	NA	Lineage 4.3	0	0	1	4398250	0.00%	4398250	97.6	99.94	0
ASM15460v2	GCA_000154605.2	SAMN00103288	PRJNA21057	6/19/12	illumina	Lineage 4.3	0	0	1	4399120	0.00%	4399120	97.6	99.94	0
ASM19407v3	GCA_000194075.3	SAMN00771609	PRJNA63839	3/14/14	illumina	M. bovis	0	0	1	4334064	0.00%	4334064	97.6	99.54	0.33
ASM35020v1	GCA_000350205.1	SAMD00061050	PRJDB66	3/1/12	sanger;roche_454;illumina	Lineage 4.1	1	0	1	4392353	0.00%	4392353	97.6	99.94	0
ASM38994v1	GCA_000389945.1	SAMN02603653	PRJNA193387	5/8/13	illumina	Lineage 4.9	0	0	1	4390306	0.00%	4390306	97.6	99.61	0
ASM104325v1	GCA_001043255.1	SAMN02864173	PRJNA251957	6/30/15	sanger;roche_454;illumina	M. bovis	0	0	1	4410431	0.00%	4410431	97.6	99.78	3
ASM154477v1	GCA_001544775.1	SAMN03257089	PRJNA270004	2/2/16	pacbio	M. bovis	0	0	1	4336227	0.00%	4336227	97.6	99.56	0
ASM207277v2	GCA_002072775.2	SAMN06622696	PRJNA379898	4/6/17	pacbio	Lineage 4.5	1	0	1	4433542	0.00%	4433542	97.6	99.94	0.42
ASM211679v1	GCA_002116795.1	SAMN05833362	PRJNA269967	5/1/17	illumina;pacbio	Lineage 2.2	1	0	1	4431885	0.00%	4431885	97.6	99.94	0.67
ASM244783v1	GCA_002447835.1	SAMN07664871	PRJNA407834	10/5/17	illumina	Lineage 4.3	0	0	1	4411327	0.00%	4411327	97.6	99.94	0

Assembly name	GenBank assembly accession	BioSample	BioProject	Date	Sequencing_technology	Lineage	Kept_analysis	Has_illumina_raw_reads	Number_of_Contigs	Total_Length_bp	Percent_Gaps	Contigs_N50	BUSCO_Completeness	CheckM_Completeness	CheckM_Contamination
ASM244787v1	GCA_002447875.1	SAMN07664873	PRJNA407834	10/5/17	illumina	Lineage 4.1	0	0	1	4411290	0.00%	4411290	97.6	99.94	0
ASM288619v1	GCA_002886195.1	SAMN08199302	PRJNA422870	1/17/18	illumina	Lineage 4.3	0	0	1	4411504	0.00%	4411504	97.6	99.94	0
ASM876167v1	GCA_008761675.1	SAMN12816907	PRJNA573545	10/1/19	illumina; pacbio	Lineage 4.4	1	1	1	4416671	0.00%	4416671	97.6	99.7	0.33
ASM1292376v1	GCA_012923765.1	SAMN13736268	PRJNA598991	4/28/20	illumina; pacbio	Lineage 8	0	1	1	4379910	0.00%	4379910	97.6	99.94	0
ASM1488462v1	GCA_014884625.1	SAMN12325246	PRJNA555636	10/13/20	pacbio	Lineage 4.3	1	0	1	4400017	0.00%	4400017	97.6	99.94	0
ASM1489946v1	GCA_014899465.1	SAMN12325267	PRJNA555636	10/20/20	pacbio	Lineage 1.2	1	0	1	4433058	0.00%	4433058	97.6	99.61	0
ASM1489956v1	GCA_014899565.1	SAMN12325262	PRJNA555636	10/20/20	pacbio	Lineage 4.4	1	0	1	4402162	0.00%	4402162	97.6	99.94	0
ASM1489960v1	GCA_014899605.1	SAMN12325260	PRJNA555636	10/20/20	pacbio	Lineage 4.3	1	0	1	4368671	0.00%	4368671	97.6	99.88	0
ASM1489988v1	GCA_014899885.1	SAMN12325245	PRJNA555636	10/20/20	pacbio	Lineage 4.3	1	0	1	4399945	0.00%	4399945	97.6	99.94	0
ASM1489990v1	GCA_014899905.1	SAMN12325244	PRJNA555636	10/20/20	pacbio	Lineage 4.3	1	0	1	4400017	0.00%	4400017	97.6	99.94	0

Assembly name	GenBank assembly accession	BioSample	BioProject	Date	Sequencing_technology	Lineage	Kept_analysis	Has_illumina_raw_reads	Number_of_Contigs	Total_Length_bp	Percent_Gaps	Contigs_N50	BUSCO_Completeness	CheckM_Completeness	CheckM_Contamination
ASM1489994v1	GCA_014899945.1	SAMN12325242	PRJNA555636	10/20/20	pacbio	Lineage 4.3	1	0	1	4400007	0.00%	4400007	97.6	99.94	0
ASM1490007v1	GCA_014900075.1	SAMN12325237	PRJNA555636	10/20/20	pacbio	Lineage 2.2	1	0	1	4417769	0.00%	4417769	97.6	99.94	0
ASM1490033v1	GCA_014900335.1	SAMN12325224	PRJNA555636	10/20/20	pacbio	Lineage 4.3	1	0	1	4405183	0.00%	4405183	97.6	99.94	0
ASM1490081v1	GCA_014900815.1	SAMN12325200	PRJNA555636	10/20/20	pacbio	Lineage 2.2	1	0	1	4411261	0.00%	4411261	97.6	99.94	0
ASM2155989v1	GCA_021559895.1	SAMN15348336	PRJNA641267	1/24/22	pacbio	Lineage 5.3	0	0	1	4438262	0.00%	4438262	97.6	99.94	0
ASM858v1	GCA_000008585.1	SAMN02603992	PRJNA223	8/4/04	sanger	Lineage 2.2	1	0	1	4403837	0.00%	4403793	98.4	99.94	0
ASM944v1	GCA_000009445.1	SAMEA2272684	PRJEA18059	1/8/07	sanger	M. bovis	0	0	1	4374522	0.00%	4374522	98.4	99.78	2.67
ASM1068v1	GCA_000010685.1	SAMD00060950	PRJDA31211	3/9/09	sanger	M. bovis	0	0	1	4371711	0.00%	4371711	98.4	99.78	1.2
ASM1614v1	GCA_000016145.1	SAMN02603128	PRJNA18883	5/31/07	NA	Lineage 4.9	0	0	1	4419977	0.00%	4419977	98.4	99.94	0
ASM1692v1	GCA_000016925.1	SAMN00103286	PRJNA15642	6/7/07	NA	Lineage 4.3	0	0	1	4424435	0.00%	4424435	98.4	99.94	0

Assembly name	GenBank assembly accession	BioSample	BioProject	Date	Sequencing_technology	Lineage	Kept_analysis	Has_illumina_raw_reads	Number_of_Contigs	Total_Length_bp	Percent_Gaps	Contigs_N50	BUSCO_Completeness	CheckM_Completeness	CheckM_Contamination
ASM15368v2	GCA_000153685.2	SAMN03081423	PRJNA17353	9/11/13	sanger	Lineage 4.1	0	0	1	4408224	0.00%	4408224	98.4	99.94	0
ASM15458v2	GCA_000154585.2	SAMN03081422	PRJNA21053	4/7/11	NA	Lineage 4.3	0	0	1	4394985	0.00%	4394985	98.4	99.94	0
ASM19318v2	GCA_000193185.2	SAMN02595346	PRJNA39283	8/19/15	sanger;illumina;pacbio	Lineage 2.2	1	0	1	4418548	0.00%	4418548	98.4	99.94	0
ASM19583v2	GCA_000195835.3	SAMEA20450668	PRJNA89	1/19/17	sanger	M. bovis	0	0	1	4349904	0.00%	4349904	98.4	99.78	0
ASM19595v2	GCA_000195955.2	SAMEA3138326	PRJNA224	2/1/13	sanger	Lineage 4.9	1	0	1	4411532	0.00%	4411532	98.4	99.94	0
ASM22443v1	GCA_000224435.1	SAMN02603755	PRJNA43171	8/25/11	sanger;roche_454	Lineage 4.3	1	0	1	4398525	0.00%	4398525	98.4	99.94	0
ASM23472v1	GCA_000234725.1	SAMN02604104	PRJNA45811	11/9/11	sanger;roche_454	M. bovis	0	0	1	4350386	0.00%	4350384	98.4	99.78	1
ASM25335v1	GCA_000253355.1	SAMEA2272165	PRJEA68095	7/5/11	sanger;illumina	Lineage 6.1	0	0	1	4389314	0.00%	4389314	98.4	99.94	0
ASM27773v2	GCA_000277735.2	SAMN03081436	PRJNA37301	11/26/13	sanger;roche_454;illumina	Lineage 4.9	1	0	1	4411709	0.00%	4411709	98.4	99.94	0
ASM33144v1	GCA_000331445.1	SAMEA2272730	PRJEA82273	1/8/13	sanger;roche_454	Lineage 4.1	1	0	1	4421197	0.00%	4421197	98.4	99.94	0

Assembly name	GenBank assembly accession	BioSample	BioProject	Date	Sequencing_technology	Lineage	Kept_analysis	Has_illumina_raw_reads	Number_of_Contigs	Total_Length_bp	Percent_Gaps	Contigs_N50	BUSCO_Completeness	CheckM_Completeness	CheckM_Contamination
ASM33871v2	GCA_000338715.2	SAMN02603488	PRJNA170028	3/8/13	roche_454;illumina	M. bovis	0	0	1	4376711	0.00%	4376711	98.4	99.78	2
ASM40061v1	GCA_000400615.1	SAMN02603646	PRJNA66069	5/20/13	roche_454;illumina	Lineage 2.2	1	0	1	4414325	0.00%	4414325	98.4	99.94	0
ASM42212v1	GCA_000422125.1	SAMN02603180	PRJNA210717	7/11/13	illumina	Lineage 1.1	0	0	1	4391174	0.00%	4391174	98.4	99.94	0
ASM57212v1	GCA_000572125.1	SAMN03081429	PRJNA66061	2/18/14	NA	Lineage 2.2	0	0	1	4407929	0.00%	4407929	98.4	99.94	0
ASM57215v1	GCA_000572155.1	SAMN03081430	PRJNA66063	2/18/14	NA	Lineage 2.2	0	0	1	4401899	0.00%	4401899	98.4	99.94	0
ASM57217v1	GCA_000572175.1	SAMN03081431	PRJNA66065	2/18/14	NA	Lineage 2.2	0	0	1	4399405	0.00%	4399405	98.4	99.94	0
ASM57219v1	GCA_000572195.1	SAMN03081432	PRJNA66071	2/18/14	NA	Lineage 2.2	0	0	1	4414346	0.00%	4414346	98.4	99.94	0
ASM69847v1	GCA_000698475.1	SAMN02803802	PRJNA178919	6/5/14	sanger;illumina	Lineage 2.2	1	0	1	4385518	0.00%	4385518	98.4	99.94	0
ASM73844v1	GCA_000738445.1	SAMN02943514	PRJNA256478	8/7/14	ion_torrent	Lineage 4.4	0	0	1	4411507	0.00%	4411507	98.4	99.94	0
ASM73847v1	GCA_000738475.1	SAMN02943515	PRJNA256478	8/7/14	ion_torrent	Lineage 4.4	0	0	1	4411515	0.00%	4411515	98.4	99.94	0

Assembly name	GenBank assembly accession	BioSample	BioProject	Date	Sequencing_technology	Lineage	Kept_analysis	Has_illumina_raw_reads	Number_of_Contigs	Total_Length_bp	Percent_Gaps	Contigs_N50	BUSCO_Completeness	CheckM_Completeness	CheckM_Contamination
ASM75652v1	GCA_000756525.1	SAMN02887314	PRJNA253748	9/22/14	roche_454	Lineage 2.2	0	0	1	4379376	0.00%	4379376	98.4	99.61	0
ASM75654v1	GCA_000756545.1	SAMN02874015	PRJNA253748	9/22/14	sanger;roche_454;illumina	Lineage 1.2	1	0	1	4410945	0.00%	4410945	98.4	99.94	0
MT49-02	GCA_000786505.1	SAMEA3865285	PRJEB4916	11/3/14	NA	Lineage 2.2	0	0	1	4412379	0.00%	4412379	98.4	99.94	0
ASM82708v1	GCA_000827085.1	SAMN03287041	PRJNA232612	1/21/15	illumina	Lineage 4.9	0	0	1	4410911	0.00%	4410911	98.4	99.94	0
ASM82899v1	GCA_000828995.1	SAMD00000334	PRJDB1582	12/23/14	pacbio	Lineage 4.9	1	0	1	4415078	0.00%	4415078	98.4	99.94	0
ASM93432v3	GCA_000934325.3	SAMN03288261	PRJNA273066	8/5/16	illumina	M. bovis	0	0	1	4347648	0.00%	4347648	98.4	99.78	0
ASM96728v1	GCA_000967285.1	SAMEA4532327	PRJNA18279	7/25/11	sanger	Lineage 4.9	0	0	1	4340116	0.00%	4340115	98.4	99.78	0.6
ASM107861v1	GCA_001078615.1	SAMN03761171	PRJNA285833	7/17/15	roche_454;illumina;pacbio	M. bovis	0	0	1	4351712	0.00%	4351712	98.4	99.78	0
ASM127455v1	GCA_001274555.1	SAMN02940916	PRJNA256163	8/26/15	sanger;roche_454	M. bovis	0	0	1	4370138	0.00%	4370138	98.4	99.78	1.2
ASM127556v2	GCA_001275565.2	SAMN03921880	PRJNA291131	8/12/16	ion_torrent;illumina	Lineage 2.2	1	0	1	4411829	0.00%	4411829	98.4	99.94	0

Assembly name	GenBank assembly accession	BioSample	BioProject	Date	Sequencing_technology	Lineage	Kept_analysis	Has_illumina_raw_reads	Number_of_Contigs	Total_Length_bp	Percent_Gaps	Contigs_N50	BUSCO_Completeness	CheckM_Completeness	CheckM_Contamination
ASM148390v1	GCA_001483905.1	SAMN04364424	PRJNA306822	1/4/16	sanger;roche_454;IonTorrent	M. bovis	0	0	1	4370705	0.00%	4370705	98.4	99.78	1.2
ASM154470v1	GCA_001544705.1	SAMN03253058	PRJNA270004	2/2/16	pacbio	Lineage 4.9	1	0	1	4421903	0.00%	4421903	98.4	99.61	0
ASM154495v1	GCA_001544955.1	SAMN03257094	PRJNA270004	2/2/16	pacbio	Lineage 4.5	1	0	1	4401829	0.00%	4401829	98.4	99.58	0
ASM158038v1	GCA_001580385.1	SAMN04517448	PRJNA313242	3/7/16	illumina	M. bovis	0	0	1	4371707	0.00%	4371707	98.4	99.78	1.2
ASM159764v2	GCA_001597645.2	SAMN04492771	PRJNA312026	12/11/19	sanger;roche_454;illumina	Lineage 4.9	0	0	1	4414742	0.00%	4414742	98.4	99.94	0
ASM170243v1	GCA_001702435.1	SAMN05447655	PRJNA335538	8/10/16	illumina	Lineage 2.2	0	0	1	4406628	0.00%	4406628	98.4	99.94	0
ASM170826v1	GCA_001708265.1	SAMN05447658	PRJNA335537	8/19/16	illumina	Lineage 2.2	0	0	1	4439387	0.00%	4439387	98.4	99.94	0
ASM175086v1	GCA_001750865.1	SAMN03301665	PRJNA273899	10/3/16	sanger;illumina	Lineage 2.2	1	0	1	4378588	0.00%	4378588	98.4	99.94	0
ASM185525v1	GCA_001855255.1	SAMN04293604	PRJNA304069	10/27/16	sanger;roche_454	Lineage 2.2	1	0	1	4402033	0.00%	4402033	98.4	99.94	0
ASM187014v1	GCA_001870145.1	SAMN05933692	PRJNA349996	11/7/16	pacbio	Lineage 2.2	1	0	1	4425860	0.00%	4425860	98.4	99.94	0.17

Assembly name	GenBank assembly accession	BioSample	BioProject	Date	Sequencing_technology	Lineage	Kept_analysis	Has_illumina_raw_reads	Number_of_Contigs	Total_Length_bp	Percent_Gaps	Contigs_N50	BUSCO_Completeness	CheckM_Completeness	CheckM_Contamination
ASM189576v1	GCA_001895765.1	SAMN06010135	PRJNA343736	12/9/16	illumina	Lineage 4.3	0	0	1	4386132	0.00%	4386132	98.4	99.94	0
ASM189578v1	GCA_001895785.1	SAMN06010493	PRJNA343736	12/9/16	illumina	Lineage 4.3	0	0	1	4359825	0.00%	4359825	98.4	99.94	0
ASM189580v1	GCA_001895805.1	SAMN06010122	PRJNA343736	12/9/16	illumina	Lineage 4.3	0	0	1	4365724	0.00%	4365722	98.4	99.94	0
ASM189582v1	GCA_001895825.1	SAMN06010171	PRJNA343736	12/9/16	illumina	Lineage 4.1	0	0	1	4376067	0.00%	4376066	98.4	99.94	0
ASM189584v1	GCA_001895845.1	SAMN06010490	PRJNA343736	12/9/16	illumina	Lineage 4.3	0	0	1	4385578	0.00%	4385578	98.4	99.94	0
ASM189586v1	GCA_001895865.1	SAMN06010343	PRJNA343736	12/9/16	illumina	Lineage 4.3	0	0	1	4386447	0.00%	4386447	98.4	99.94	0
ASM192248v1	GCA_001922485.1	SAMN06141171	PRJNA357378	12/27/16	illumina	Lineage 4.5	0	0	1	4411511	0.00%	4411511	98.4	99.94	0
ASM193872v1	GCA_001938725.1	SAMN05414673	PRJNA329548	1/6/17	pacbio	Lineage 4.9	1	0	1	4426109	0.00%	4426109	98.4	99.94	0
ASM211675v1	GCA_002116755.1	SAMN05833360	PRJNA269967	5/1/17	illumina; pacbio	Lineage 2.2	1	0	1	4427062	0.00%	4427062	98.4	99.94	0.33
ASM211677v1	GCA_002116775.1	SAMN05833361	PRJNA269967	5/1/17	illumina; pacbio	Lineage 2.2	1	0	1	4441591	0.00%	4441591	98.4	99.94	1

Assembly name	GenBank assembly accession	BioSample	BioProject	Date	Sequencing_technology	Lineage	Kept_analysis	Has_illumina_raw_reads	Number_of_Contigs	Total_Length_bp	Percent_Gaps	Contigs_N50	BUSCO_Completeness	CheckM_Completeness	CheckM_Contamination
ASM211681v1	GCA_002116815.1	SAMN05833364	PRJNA269967	5/1/17	illumina; pacbio	Lineage 2.2	1	0	1	4406925	0.00%	4406925	98.4	99.94	0
ASM211683v1	GCA_002116835.1	SAMN05833363	PRJNA269967	5/1/17	illumina; pacbio	Lineage 2.2	1	0	1	4444417	0.00%	4444417	98.4	99.94	0.67
ASM211685v1	GCA_002116855.1	SAMN05833359	PRJNA269967	5/1/17	illumina; pacbio	Lineage 2.2	1	0	1	4380156	0.00%	4380156	98.4	99.94	0.33
ASM220823v1	GCA_002208235.1	SAMN06622697	PRJNA379898	6/26/17	pacbio	Lineage 2.2	1	0	1	4417716	0.00%	4417716	98.4	99.94	0
ASM235601v1	GCA_002356015.1	SAMD00065354	PRJDB5220	12/1/16	pacbio	Lineage 4.3	1	0	1	4380602	0.00%	4380602	98.4	99.94	0
ASM235625v1	GCA_002356255.1	SAMD00076259	PRJDB5608	4/25/17	sanger;illumina;pacbio	Lineage 1.1	1	0	1	4399916	0.00%	4399916	98.4	99.94	0
ASM235793v1	GCA_002357935.1	SAMD00076256	PRJDB5607	5/10/17	sanger;illumina;pacbio	Lineage 2.2	1	0	1	4411033	0.00%	4411033	98.4	99.94	0
ASM235795v1	GCA_002357955.1	SAMD00076257	PRJDB5607	5/10/17	sanger;illumina;pacbio	Lineage 2.2	1	0	1	4421540	0.00%	4421540	98.4	99.94	0
ASM235797v1	GCA_002357975.1	SAMD00076258	PRJDB5607	5/10/17	sanger;illumina;pacbio	Lineage 2.2	1	0	1	4413362	0.00%	4413362	98.4	99.94	0
ASM244687v1	GCA_002446875.1	SAMN07664823	PRJNA407834	10/5/17	illumina	Lineage 4.3	0	0	1	4411288	0.00%	4411288	98.4	99.94	0

Assembly name	GenBank assembly accession	BioSample	BioProject	Date	Sequencing_technology	Lineage	Kept_analysis	Has_illumina_raw_reads	Number_of_Contigs	Total_Length_bp	Percent_Gaps	Contigs_N50	BUSCO_Completeness	CheckM_Completeness	CheckM_Contamination
ASM244689v1	GCA_002446895.1	SAMN07664824	PRJNA407834	10/5/17	illumina	Lineage 4.3	0	0	1	4411271	0.00%	4411271	98.4	99.94	0
ASM244691v1	GCA_002446915.1	SAMN07664825	PRJNA407834	10/5/17	illumina	Lineage 4	0	0	1	4411312	0.00%	4411312	98.4	99.94	0
ASM244693v1	GCA_002446935.1	SAMN07664826	PRJNA407834	10/5/17	illumina	Lineage 4.1	0	0	1	4411180	0.00%	4411180	98.4	99.94	0
ASM244695v1	GCA_002446955.1	SAMN07664827	PRJNA407834	10/5/17	illumina	Lineage 4.1	0	0	1	4411382	0.00%	4411382	98.4	99.94	0
ASM244697v1	GCA_002446975.1	SAMN07664828	PRJNA407834	10/5/17	illumina	Lineage 4.3	0	0	1	4411180	0.00%	4411180	98.4	99.94	0
ASM244699v1	GCA_002446995.1	SAMN07664829	PRJNA407834	10/5/17	illumina	Lineage 4.3	0	0	1	4411208	0.00%	4411208	98.4	99.94	0
ASM244701v1	GCA_002447015.1	SAMN07664830	PRJNA407834	10/5/17	illumina	Lineage 4.1	0	0	1	4411436	0.00%	4411436	98.4	99.94	0
ASM244703v1	GCA_002447035.1	SAMN07664831	PRJNA407834	10/5/17	illumina	Lineage 4.3	0	0	1	4411353	0.00%	4411353	98.4	99.94	0
ASM244705v1	GCA_002447055.1	SAMN07664832	PRJNA407834	10/5/17	illumina	Lineage 4	0	0	1	4411315	0.00%	4411315	98.4	99.94	0
ASM244707v1	GCA_002447075.1	SAMN07664833	PRJNA407834	10/5/17	illumina	Lineage 4	0	0	1	4411280	0.00%	4411280	98.4	99.94	0

Assembly name	GenBank assembly accession	BioSample	BioProject	Date	Sequencing_technology	Lineage	Kept_analysis	Has_illumina_raw_reads	Number_of_Contigs	Total_Length_bp	Percent_Gaps	Contigs_N50	BUSCO_Completeness	CheckM_Completeness	CheckM_Contamination
ASM244709v1	GCA_002447095.1	SAMN07664834	PRJNA407834	10/5/17	illumina	Lineage 4.3	0	0	1	4411215	0.00%	4411215	98.4	99.94	0
ASM244711v1	GCA_002447115.1	SAMN07664835	PRJNA407834	10/5/17	illumina	Lineage 4.3	0	0	1	4411148	0.00%	4411148	98.4	99.94	0
ASM244713v1	GCA_002447135.1	SAMN07664836	PRJNA407834	10/5/17	illumina	Lineage 2.2	0	0	1	4411121	0.00%	4411121	98.4	99.94	0
ASM244715v1	GCA_002447155.1	SAMN07664837	PRJNA407834	10/5/17	illumina	Lineage 4.3	0	0	1	4411217	0.00%	4411217	98.4	99.94	0
ASM244717v1	GCA_002447175.1	SAMN07664838	PRJNA407834	10/5/17	illumina	Lineage 4.3	0	0	1	4411143	0.00%	4411143	98.4	99.94	0
ASM244719v1	GCA_002447195.1	SAMN07664839	PRJNA407834	10/5/17	illumina	Lineage 4.3	0	0	1	4411318	0.00%	4411318	98.4	99.94	0
ASM244721v1	GCA_002447215.1	SAMN07664840	PRJNA407834	10/5/17	illumina	Lineage 4.3	0	0	1	4411331	0.00%	4411331	98.4	99.94	0
ASM244723v1	GCA_002447235.1	SAMN07664841	PRJNA407834	10/5/17	illumina	Lineage 4	0	0	1	4411398	0.00%	4411398	98.4	99.94	0
ASM244725v1	GCA_002447255.1	SAMN07664842	PRJNA407834	10/5/17	illumina	Lineage 4.3	0	0	1	4411342	0.00%	4411342	98.4	99.94	0
ASM244727v1	GCA_002447275.1	SAMN07664843	PRJNA407834	10/5/17	illumina	Lineage 4.1	0	0	1	4411408	0.00%	4411408	98.4	99.94	0

Assembly name	GenBank assembly accession	BioSample	BioProject	Date	Sequencing_technology	Lineage	Kept_analysis	Has_illumina_raw_reads	Number_of_Contigs	Total_Length_bp	Percent_Gaps	Contigs_N50	BUSCO_Completeness	CheckM_Completeness	CheckM_Contamination
ASM244729v1	GCA_002447295.1	SAMN07664844	PRJNA407834	10/5/17	illumina	Lineage 4.3	0	0	1	4411306	0.00%	4411306	98.4	99.94	0
ASM244731v1	GCA_002447315.1	SAMN07664845	PRJNA407834	10/5/17	illumina	Lineage 2.2	0	0	1	4411134	0.00%	4411134	98.4	99.94	0
ASM244733v1	GCA_002447335.1	SAMN07664846	PRJNA407834	10/5/17	illumina	Lineage 4.1	0	0	1	4411337	0.00%	4411337	98.4	99.94	0
ASM244735v1	GCA_002447355.1	SAMN07664847	PRJNA407834	10/5/17	illumina	Lineage 4.1	0	0	1	4411385	0.00%	4411385	98.4	99.94	0
ASM244737v1	GCA_002447375.1	SAMN07664848	PRJNA407834	10/5/17	illumina	Lineage 4.1	0	0	1	4411394	0.00%	4411394	98.4	99.94	0
ASM244739v1	GCA_002447395.1	SAMN07664849	PRJNA407834	10/5/17	illumina	Lineage 2.2	0	0	1	4411115	0.00%	4411115	98.4	99.94	0
ASM244741v1	GCA_002447415.1	SAMN07664850	PRJNA407834	10/5/17	illumina	Lineage 4.7	0	0	1	4411439	0.00%	4411439	98.4	99.94	0
ASM244743v1	GCA_002447435.1	SAMN07664851	PRJNA407834	10/5/17	illumina	Lineage 4.3	0	0	1	4411230	0.00%	4411230	98.4	99.94	0
ASM244745v1	GCA_002447455.1	SAMN07664852	PRJNA407834	10/5/17	illumina	Lineage 4.1	0	0	1	4411412	0.00%	4411412	98.4	99.94	0
ASM244747v1	GCA_002447475.1	SAMN07664853	PRJNA407834	10/5/17	illumina	Lineage 4	0	0	1	4411415	0.00%	4411415	98.4	99.94	0

Assembly name	GenBank assembly accession	BioSample	BioProject	Date	Sequencing_technology	Lineage	Kept_analysis	Has_illumina_raw_reads	Number_of_Contigs	Total_Length_bp	Percent_Gaps	Contigs_N50	BUSCO_Completeness	CheckM_Completeness	CheckM_Contamination
ASM244749v1	GCA_002447495.1	SAMN07664854	PRJNA407834	10/5/17	illumina	Lineage 4.3	0	0	1	4411211	0.00%	4411211	98.4	99.94	0
ASM244751v1	GCA_002447515.1	SAMN07664855	PRJNA407834	10/5/17	illumina	Lineage 4.1	0	0	1	4411475	0.00%	4411475	98.4	99.94	0
ASM244753v1	GCA_002447535.1	SAMN07664856	PRJNA407834	10/5/17	illumina	Lineage 4.3	0	0	1	4411243	0.00%	4411243	98.4	99.94	0
ASM244755v1	GCA_002447555.1	SAMN07664857	PRJNA407834	10/5/17	illumina	Lineage 4.3	0	0	1	4411137	0.00%	4411137	98.4	99.94	0
ASM244757v1	GCA_002447575.1	SAMN07664858	PRJNA407834	10/5/17	illumina	Lineage 4.8	0	0	1	4411365	0.00%	4411365	98.4	99.94	0
ASM244759v1	GCA_002447595.1	SAMN07664859	PRJNA407834	10/5/17	illumina	Lineage 2.2	0	0	1	4411186	0.00%	4411186	98.4	99.94	0
ASM244761v1	GCA_002447615.1	SAMN07664860	PRJNA407834	10/5/17	illumina	Lineage 4	0	0	1	4411340	0.00%	4411340	98.4	99.94	0
ASM244763v1	GCA_002447635.1	SAMN07664861	PRJNA407834	10/5/17	illumina	Lineage 4.3	0	0	1	4411321	0.00%	4411321	98.4	99.94	0
ASM244765v1	GCA_002447655.1	SAMN07664862	PRJNA407834	10/5/17	illumina	Lineage 4	0	0	1	4411331	0.00%	4411331	98.4	99.94	0
ASM244767v1	GCA_002447675.1	SAMN07664863	PRJNA407834	10/5/17	illumina	Lineage 4.3	0	0	1	4411326	0.00%	4411326	98.4	99.94	0

Assembly name	GenBank assembly accession	BioSample	BioProject	Date	Sequencing_technology	Lineage	Kept_analysis	Has_illumina_raw_reads	Number_of_Contigs	Total_Length_bp	Percent_Gaps	Contigs_N50	BUSCO_Completeness	CheckM_Completeness	CheckM_Contamination
ASM244769v1	GCA_002447695.1	SAMN07664864	PRJNA407834	10/5/17	illumina	Lineage 4.3	0	0	1	4411379	0.00%	4411379	98.4	99.94	0
ASM244771v1	GCA_002447715.1	SAMN07664865	PRJNA407834	10/5/17	illumina	Lineage 4.1	0	0	1	4411392	0.00%	4411392	98.4	99.94	0
ASM244773v1	GCA_002447735.1	SAMN07664866	PRJNA407834	10/5/17	illumina	Lineage 4.8	0	0	1	4411494	0.00%	4411494	98.4	99.94	0
ASM244775v1	GCA_002447755.1	SAMN07664867	PRJNA407834	10/5/17	illumina	Lineage 4.3	0	0	1	4411335	0.00%	4411335	98.4	99.94	0
ASM244777v1	GCA_002447775.1	SAMN07664868	PRJNA407834	10/5/17	illumina	Lineage 4.1	0	0	1	4411449	0.00%	4411449	98.4	99.94	0
ASM244779v1	GCA_002447795.1	SAMN07664869	PRJNA407834	10/5/17	illumina	Lineage 4.1	0	0	1	4411392	0.00%	4411392	98.4	99.94	0
ASM244781v1	GCA_002447815.1	SAMN07664870	PRJNA407834	10/5/17	illumina	Lineage 4.3	0	0	1	4411299	0.00%	4411299	98.4	99.94	0.07
ASM244785v1	GCA_002447855.1	SAMN07664872	PRJNA407834	10/5/17	illumina	Lineage 4.3	0	0	1	4411315	0.00%	4411315	98.4	99.94	0
ASM244789v1	GCA_002447895.1	SAMN07664874	PRJNA407834	10/5/17	illumina	Lineage 4.3	0	0	1	4411338	0.00%	4411338	98.4	99.94	0
ASM244791v1	GCA_002447915.1	SAMN07664875	PRJNA407834	10/5/17	illumina	Lineage 4.1	0	0	1	4411432	0.00%	4411432	98.4	99.94	0

Assembly name	GenBank assembly accession	BioSample	BioProject	Date	Sequencing_technology	Lineage	Kept_analysis	Has_illumina_raw_reads	Number_of_Contigs	Total_Length_bp	Percent_Gaps	Contigs_N50	BUSCO_Completeness	CheckM_Completeness	CheckM_Contamination
ASM244793v1	GCA_002447935.1	SAMN07664876	PRJNA407834	10/5/17	illumina	Lineage 2.2	0	0	1	4411159	0.00%	4411159	98.4	99.94	0
ASM244795v1	GCA_002447955.1	SAMN07664877	PRJNA407834	10/5/17	illumina	Lineage 4.3	0	0	1	4411229	0.00%	4411229	98.4	99.94	0
ASM244797v1	GCA_002447975.1	SAMN07664878	PRJNA407834	10/5/17	illumina	Lineage 4.1	0	0	1	4411479	0.00%	4411479	98.4	99.94	0
ASM244799v1	GCA_002447995.1	SAMN07664879	PRJNA407834	10/5/17	illumina	Lineage 4.3	0	0	1	4411352	0.00%	4411352	98.4	99.94	0
ASM244801v1	GCA_002448015.1	SAMN07664880	PRJNA407834	10/5/17	illumina	Lineage 4.7	0	0	1	4411454	0.00%	4411454	98.4	99.94	0
ASM244803v1	GCA_002448035.1	SAMN07664881	PRJNA407834	10/5/17	illumina	Lineage 4.1	0	0	1	4411157	0.00%	4411157	98.4	99.94	0
ASM244805v1	GCA_002448055.1	SAMN07664882	PRJNA407834	10/5/17	illumina	Lineage 4	0	0	1	4411316	0.00%	4411316	98.4	99.94	0
ASM244807v1	GCA_002448075.1	SAMN07664883	PRJNA407834	10/5/17	illumina	Lineage 4.3	0	0	1	4411284	0.00%	4411284	98.4	99.94	0
ASM244809v1	GCA_002448095.1	SAMN07664884	PRJNA407834	10/5/17	illumina	Lineage 4.8	0	0	1	4411314	0.00%	4411314	98.4	99.94	0
ASM244811v1	GCA_002448115.1	SAMN07664885	PRJNA407834	10/5/17	illumina	Lineage 4.3	0	0	1	4411369	0.00%	4411369	98.4	99.94	0

Assembly name	GenBank assembly accession	BioSample	BioProject	Date	Sequencing_technology	Lineage	Kept_analysis	Has_illumina_raw_reads	Number_of_Contigs	Total_Length_bp	Percent_Gaps	Contigs_N50	BUSCO_Completeness	CheckM_Completeness	CheckM_Contamination
ASM244813v1	GCA_002448135.1	SAMN07664886	PRJNA407834	10/5/17	illumina	Lineage 4.1	0	0	1	4411457	0.00%	4411457	98.4	99.94	0
ASM244815v1	GCA_002448155.1	SAMN07664887	PRJNA407834	10/5/17	illumina	Lineage 4.1	0	0	1	4411442	0.00%	4411442	98.4	99.94	0
ASM244817v1	GCA_002448175.1	SAMN07664888	PRJNA407834	10/5/17	illumina	Lineage 4.3	0	0	1	4411310	0.00%	4411310	98.4	99.94	0
ASM244819v1	GCA_002448195.1	SAMN07664889	PRJNA407834	10/5/17	illumina	Lineage 2.2	0	0	1	4411173	0.00%	4411173	98.4	99.94	0
ASM244821v1	GCA_002448215.1	SAMN07664890	PRJNA407834	10/5/17	illumina	Lineage 4.1	0	0	1	4411414	0.00%	4411414	98.4	99.94	0
ASM288614v1	GCA_002886145.1	SAMN08199254	PRJNA422870	1/17/18	illumina	Lineage 4.1	0	0	1	4411563	0.00%	4411563	98.4	99.94	0
ASM288616v1	GCA_002886165.1	SAMN08199260	PRJNA422870	1/17/18	illumina	Lineage 4.8	0	0	1	4411442	0.00%	4411442	98.4	99.94	0
ASM288622v1	GCA_002886225.1	SAMN08199276	PRJNA422870	1/17/18	illumina	Lineage 4.3	0	0	1	4411508	0.00%	4411508	98.4	99.94	0
ASM288633v1	GCA_002886335.1	SAMN08199305	PRJNA422870	1/17/18	illumina	Lineage 4.3	0	0	1	4411602	0.00%	4411602	98.4	99.94	0
ASM288640v1	GCA_002886405.1	SAMN08199301	PRJNA422870	1/17/18	illumina	Lineage 4.1	0	0	1	4411413	0.00%	4411413	98.4	99.94	0

Assembly name	GenBank assembly accession	BioSample	BioProject	Date	Sequencing_technology	Lineage	Kept_analysis	Has_illumina_raw_reads	Number_of_Contigs	Total_Length_bp	Percent_Gaps	Contigs_N50	BUSCO_Completeness	CheckM_Completeness	CheckM_Contamination
ASM288650v1	GCA_002886505.1	SAMN08199277	PRJNA422870	1/17/18	illumina	Lineage 4.3	0	0	1	4411399	0.00%	4411399	98.4	99.94	0
ASM288658v1	GCA_002886585.1	SAMN08199266	PRJNA422870	1/17/18	illumina	Lineage 4.3	0	0	1	4411519	0.00%	4411519	98.4	99.94	0
ASM288668v1	GCA_002886685.1	SAMN08199347	PRJNA422870	1/17/18	illumina	Lineage 4.3	0	0	1	4411526	0.00%	4411526	98.4	99.94	0
ASM288677v1	GCA_002886775.1	SAMN08199271	PRJNA422870	1/17/18	illumina	Lineage 4.3	0	0	1	4411510	0.00%	4411510	98.4	99.94	0
ASM288686v1	GCA_002886865.1	SAMN08199268	PRJNA422870	1/17/18	illumina	Lineage 4.1	0	0	1	4411478	0.00%	4411478	98.4	99.94	0
ASM288694v1	GCA_002886945.1	SAMN08199265	PRJNA422870	1/17/18	illumina	Lineage 4.1	0	0	1	4411443	0.00%	4411443	98.4	99.94	0
ASM288706v1	GCA_002887065.1	SAMN08199267	PRJNA422870	1/17/18	illumina	Lineage 4.8	0	0	1	4411469	0.00%	4411469	98.4	99.94	0
ASM288714v1	GCA_002887145.1	SAMN08199303	PRJNA422870	1/17/18	illumina	Lineage 4.8	0	0	1	4411469	0.00%	4411469	98.4	99.94	0
ASM288725v1	GCA_002887255.1	SAMN08199306	PRJNA422870	1/17/18	illumina	Lineage 4.3	0	0	1	4411463	0.00%	4411463	98.4	99.94	0
ASM288733v1	GCA_002887335.1	SAMN08199348	PRJNA422870	1/17/18	illumina	Lineage 4.3	0	0	1	4411446	0.00%	4411446	98.4	99.94	0

Assembly name	GenBank assembly accession	BioSample	BioProject	Date	Sequencing_technology	Lineage	Kept_analysis	Has_illumina_raw_reads	Number_of_Contigs	Total_Length_bp	Percent_Gaps	Contigs_N50	BUSCO_Completeness	CheckM_Completeness	CheckM_Contamination
ASM297547v1	GCA_002975475.1	SAMN08321034	PRJNA428763	3/7/18	illumina; pacbio	M. bovis	0	0	1	4355637	0.00%	4355637	98.4	99.78	0.17
ASM300611v1	GCA_003006115.1	SAMN07414820	PRJNA395787	3/16/18	pacbio	Lineage 2.2	1	0	1	4413669	0.00%	4413669	98.4	99.94	0
ASM300613v1	GCA_003006135.1	SAMN07414819	PRJNA395786	3/16/18	pacbio	Lineage 2.2	1	0	1	4413712	0.00%	4413712	98.4	99.94	0
ASM326500v1	GCA_003265005.1	SAMN09453176	PRJNA476697	6/26/18	illumina	Lineage 2.2	0	0	1	4418559	0.00%	4418559	98.4	99.94	0
ASM328712v1	GCA_003287125.1	SAMN06292592	PRJNA369711	7/3/18	pacbio	Lineage 2.2	1	0	1	4416938	0.00%	4416938	98.4	99.94	0
ASM328714v1	GCA_003287145.1	SAMN06292590	PRJNA369711	7/3/18	pacbio	Lineage 4.1	1	0	1	4413214	0.00%	4413214	98.4	99.94	0
ASM328716v1	GCA_003287165.1	SAMN06292065	PRJNA369711	7/3/18	pacbio	Lineage 4.5	1	0	1	4418796	0.00%	4418796	98.4	99.94	0
ASM328718v1	GCA_003287185.1	SAMN06292067	PRJNA369711	7/3/18	pacbio	Lineage 2.2	1	0	1	4406346	0.00%	4406346	98.4	99.94	0
ASM329365v1	GCA_003293655.1	SAMN08973501	PRJNA453104	7/9/18	illumina	Lineage 1.2	0	0	1	4359659	0.00%	4359659	98.4	99.94	0
ASM515578v1	GCA_005155785.1	SAMN10219709	PRJNA494982	5/6/19	illumina; pacbio	M. bovis	0	1	1	4332698	0.00%	4332698	98.4	99.78	0.67

Assembly name	GenBank assembly accession	BioSample	BioProject	Date	Sequencing_technology	Lineage	Kept_analysis	Has_illumina_raw_reads	Number_of_Contigs	Total_Length_bp	Percent_Gaps	Contigs_N50	BUSCO_Completeness	CheckM_Completeness	CheckM_Contamination
ASM515610v1	GCA_005156105.1	SAMN10219708	PRJNA494982	5/6/19	illumina; pacbio	M. bovis	0	1	1	4411814	0.00%	4411814	98.4	99.78	3
ASM783393v1	GCA_007833935.1	SAMN12126281	PRJNA550393	8/5/19	pacbio	Lineage 4.1	1	0	1	4426525	0.00%	4426525	98.4	99.94	0
ASM966487v1	GCA_009664875.1	SAMN13191639	PRJNA556438	11/17/19	illumina; pacbio	Lineage 1.1	1	1	1	4414769	0.00%	4414769	98.4	99.94	0
ASM973021v1	GCA_009730215.1	SAMN11056466	PRJNA231221	12/5/19	illumina; pacbio	Lineage 1.2	1	1	1	4441988	0.00%	4441988	98.4	99.94	0
ASM973023v1	GCA_009730235.1	SAMN11056465	PRJNA231221	12/5/19	illumina; pacbio	Lineage 3	1	1	1	4434666	0.00%	4434666	98.4	99.94	0
ASM976267v2	GCA_009762675.2	SAMN10417149	PRJNA505382	2/24/20	illumina; pacbio	Lineage 2.2	1	1	1	4418417	0.00%	4418417	98.4	99.78	0
ASM1301038v1	GCA_013010385.1	SAMN14598146	PRJNA625312	5/11/20	illumina; nanopore	Lineage 4.3	1	0	1	4394156	0.00%	4394156	98.4	99.94	0
ASM1326763v1	GCA_013267635.1	SAMN11056472	PRJNA231221	6/4/20	illumina; pacbio	Lineage 4.9	1	1	1	4417931	0.00%	4417931	98.4	99.94	0
ASM1326765v1	GCA_013267655.1	SAMN11056471	PRJNA231221	6/4/20	illumina; pacbio	Lineage 2.2	1	1	1	4414577	0.00%	4414577	98.4	99.94	0.17
ASM1488464v1	GCA_014884645.1	SAMN12325211	PRJNA555636	10/13/20	pacbio	Lineage 2.2	1	0	1	4421012	0.00%	4421012	98.4	99.94	0.67

Assembly name	GenBank assembly accession	BioSample	BioProject	Date	Sequencing_technology	Lineage	Kept_analysis	Has_illumina_raw_reads	Number_of_Contigs	Total_Length_bp	Percent_Gaps	Contigs_N50	BUSCO_Completeness	CheckM_Completeness	CheckM_Contamination
ASM1488466v1	GCA_014884665.1	SAMN12325208	PRJNA555636	10/13/20	pacbio	Lineage 2.2	1	0	1	4416715	0.00%	4416715	98.4	99.94	0
ASM1489920v1	GCA_014899205.1	SAMN12325280	PRJNA555636	10/20/20	pacbio	Lineage 4.1	1	0	1	4405198	0.00%	4405198	98.4	99.94	0
ASM1489922v1	GCA_014899225.1	SAMN12325279	PRJNA555636	10/20/20	pacbio	Lineage 2.2	1	0	1	4415138	0.00%	4415138	98.4	99.94	0
ASM1489924v1	GCA_014899245.1	SAMN12325278	PRJNA555636	10/20/20	pacbio	Lineage 1.1	1	0	1	4420315	0.00%	4420315	98.4	99.94	0
ASM1489926v1	GCA_014899265.1	SAMN12325276	PRJNA555636	10/20/20	pacbio	Lineage 1.1	1	0	1	4412837	0.00%	4412837	98.4	99.94	0
ASM1489928v1	GCA_014899285.1	SAMN12325277	PRJNA555636	10/20/20	pacbio	Lineage 1.1	1	0	1	4420316	0.00%	4420316	98.4	99.94	0
ASM1489930v1	GCA_014899305.1	SAMN12325275	PRJNA555636	10/20/20	pacbio	Lineage 1.1	1	0	1	4431110	0.00%	4431110	98.4	99.94	0
ASM1489932v1	GCA_014899325.1	SAMN12325274	PRJNA555636	10/20/20	pacbio	Lineage 1.1	1	0	1	4407803	0.00%	4407803	98.4	99.94	0
ASM1489934v1	GCA_014899345.1	SAMN12325273	PRJNA555636	10/20/20	pacbio	Lineage 1.1	1	0	1	4408229	0.00%	4408229	98.4	99.94	0
ASM1489936v1	GCA_014899365.1	SAMN12325272	PRJNA555636	10/20/20	pacbio	Lineage 1.2	1	0	1	4426614	0.00%	4426614	98.4	99.61	0

Assembly name	GenBank assembly accession	BioSample	BioProject	Date	Sequencing_technology	Lineage	Kept_analysis	Has_illumina_raw_reads	Number_of_Contigs	Total_Length_bp	Percent_Gaps	Contigs_N50	BUSCO_Completeness	CheckM_Completeness	CheckM_Contamination
ASM1489938v1	GCA_014899385.1	SAMN12325271	PRJNA555636	10/20/20	pacbio	Lineage 2.1	1	0	1	4397372	0.00%	4397372	98.4	99.94	0
ASM1489940v1	GCA_014899405.1	SAMN12325270	PRJNA555636	10/20/20	pacbio	Lineage 1.1	1	0	1	4410956	0.00%	4410956	98.4	99.94	0
ASM1489942v1	GCA_014899425.1	SAMN12325269	PRJNA555636	10/20/20	pacbio	Lineage 2.2	1	0	1	4406587	0.00%	4406587	98.4	99.94	0
ASM1489944v1	GCA_014899445.1	SAMN12325268	PRJNA555636	10/20/20	pacbio	Lineage 1.2	1	0	1	4427826	0.00%	4427826	98.4	99.94	0
ASM1489948v1	GCA_014899485.1	SAMN12325266	PRJNA555636	10/20/20	pacbio	Lineage 1.1	1	0	1	4410647	0.00%	4410647	98.4	99.94	0
ASM1489950v1	GCA_014899505.1	SAMN12325265	PRJNA555636	10/20/20	pacbio	Lineage 7	0	0	1	4410029	0.00%	4410029	98.4	99.94	0
ASM1489952v1	GCA_014899525.1	SAMN12325264	PRJNA555636	10/20/20	pacbio	Lineage 4.8	1	0	1	4406516	0.00%	4406516	98.4	99.94	0
ASM1489954v1	GCA_014899545.1	SAMN12325263	PRJNA555636	10/20/20	pacbio	Lineage 4.1	1	0	1	4402473	0.00%	4402473	98.4	99.94	0
ASM1489958v1	GCA_014899585.1	SAMN12325261	PRJNA555636	10/20/20	pacbio	Lineage 4.8	1	0	1	4397139	0.00%	4397139	98.4	99.94	0
ASM1489962v1	GCA_014899625.1	SAMN12325259	PRJNA555636	10/20/20	pacbio	Lineage 4.7	1	0	1	4391433	0.00%	4391433	98.4	99.94	0

Assembly name	GenBank assembly accession	BioSample	BioProject	Date	Sequencing_technology	Lineage	Kept_analysis	Has_illumina_raw_reads	Number_of_Contigs	Total_Length_bp	Percent_Gaps	Contigs_N50	BUSCO_Completeness	CheckM_Completeness	CheckM_Contamination
ASM1489964v1	GCA_014899645.1	SAMN12325258	PRJNA555636	10/20/20	pacbio	Lineage 4.3	1	0	1	4408888	0.00%	4408888	98.4	99.94	0
ASM1489966v1	GCA_014899665.1	SAMN12325257	PRJNA555636	10/20/20	pacbio	Lineage 1.2	1	0	1	4413498	0.00%	4413498	98.4	99.94	0
ASM1489968v1	GCA_014899685.1	SAMN12325256	PRJNA555636	10/20/20	pacbio	Lineage 4.3	1	0	1	4407077	0.00%	4407077	98.4	99.94	0
ASM1489970v1	GCA_014899705.1	SAMN12325255	PRJNA555636	10/20/20	pacbio	Lineage 3.1	1	0	1	4438102	0.00%	4438102	98.4	99.94	0
ASM1489972v1	GCA_014899725.1	SAMN12325254	PRJNA555636	10/20/20	pacbio	Lineage 4.1	1	0	1	4408176	0.00%	4408176	98.4	99.61	0
ASM1489974v1	GCA_014899745.1	SAMN12325253	PRJNA555636	10/20/20	pacbio	Lineage 2.2	1	0	1	4416761	0.00%	4416761	98.4	99.94	0
ASM1489976v1	GCA_014899765.1	SAMN12325252	PRJNA555636	10/20/20	pacbio	Lineage 4.1	1	0	1	4424310	0.00%	4424310	98.4	99.53	0
ASM1489978v1	GCA_014899785.1	SAMN12325251	PRJNA555636	10/20/20	pacbio	Lineage 2.2	1	0	1	4408117	0.00%	4408117	98.4	99.94	0
ASM1489980v1	GCA_014899805.1	SAMN12325250	PRJNA555636	10/20/20	pacbio	Lineage 4.1	1	0	1	4428457	0.00%	4428457	98.4	99.94	0
ASM1489982v1	GCA_014899825.1	SAMN12325249	PRJNA555636	10/20/20	pacbio	Lineage 2.2	1	0	1	4401396	0.00%	4401396	98.4	99.94	0

Assembly name	GenBank assembly accession	BioSample	BioProject	Date	Sequencing_technology	Lineage	Kept_analysis	Has_illumina_raw_reads	Number_of_Contigs	Total_Length_bp	Percent_Gaps	Contigs_N50	BUSCO_Completeness	CheckM_Completeness	CheckM_Contamination
ASM1489984v1	GCA_014899845.1	SAMN12325248	PRJNA555636	10/20/20	pacbio	Lineage 4.3	1	0	1	4414542	0.00%	4414542	98.4	99.94	0
ASM1489986v1	GCA_014899865.1	SAMN12325247	PRJNA555636	10/20/20	pacbio	Lineage 2.2	1	0	1	4399842	0.00%	4399842	98.4	99.94	0
ASM1489992v1	GCA_014899925.1	SAMN12325243	PRJNA555636	10/20/20	pacbio	Lineage 4.1	1	0	1	4401614	0.00%	4401614	98.4	99.94	0
ASM1489998v1	GCA_014899985.1	SAMN12325240	PRJNA555636	10/20/20	pacbio	Lineage 1.2	1	0	1	4450340	0.00%	4450340	98.4	99.94	0
ASM1490000v1	GCA_014900005.1	SAMN12325239	PRJNA555636	10/20/20	pacbio	Lineage 1.2	1	0	1	4433503	0.00%	4433503	98.4	99.94	0
ASM1490005v1	GCA_014900055.1	SAMN12325238	PRJNA555636	10/20/20	pacbio	Lineage 4.3	1	0	1	4376753	0.00%	4376753	98.4	99.94	0
ASM1490009v1	GCA_014900095.1	SAMN12325236	PRJNA555636	10/20/20	pacbio	Lineage 4.2	1	0	1	4425172	0.00%	4425172	98.4	99.94	0
ASM1490011v1	GCA_014900115.1	SAMN12325235	PRJNA555636	10/20/20	pacbio	Lineage 2.2	1	0	1	4404274	0.00%	4404274	98.4	99.94	0
ASM1490013v1	GCA_014900135.1	SAMN12325234	PRJNA555636	10/20/20	pacbio	Lineage 4.2	1	0	1	4438223	0.00%	4438223	98.4	99.94	0
ASM1490015v1	GCA_014900155.1	SAMN12325233	PRJNA555636	10/20/20	pacbio	Lineage 4.1	1	0	1	4409038	0.00%	4409038	98.4	99.94	0

Assembly name	GenBank assembly accession	BioSample	BioProject	Date	Sequencing_technology	Lineage	Kept_analysis	Has_illumina_raw_reads	Number_of_Contigs	Total_Length_bp	Percent_Gaps	Contigs_N50	BUSCO_Completeness	CheckM_Completeness	CheckM_Contamination
ASM1490017v1	GCA_014900175.1	SAMN12325232	PRJNA555636	10/20/20	pacbio	Lineage 2.2	1	0	1	4410873	0.00%	4410873	98.4	99.94	0
ASM1490019v1	GCA_014900195.1	SAMN12325231	PRJNA555636	10/20/20	pacbio	Lineage 2.2	1	0	1	4418552	0.00%	4418552	98.4	99.28	0
ASM1490021v1	GCA_014900215.1	SAMN12325230	PRJNA555636	10/20/20	pacbio	Lineage 4.2	1	0	1	4438363	0.00%	4438363	98.4	99.9	0
ASM1490023v1	GCA_014900235.1	SAMN12325229	PRJNA555636	10/20/20	pacbio	Lineage 4.2	1	0	1	4438281	0.00%	4438281	98.4	99.61	0
ASM1490025v1	GCA_014900255.1	SAMN12325228	PRJNA555636	10/20/20	pacbio	Lineage 4.2	1	0	1	4438366	0.00%	4438366	98.4	99.94	0
ASM1490027v1	GCA_014900275.1	SAMN12325227	PRJNA555636	10/20/20	pacbio	Lineage 4.2	1	0	1	4438339	0.00%	4438339	98.4	99.94	0
ASM1490029v1	GCA_014900295.1	SAMN12325226	PRJNA555636	10/20/20	pacbio	Lineage 4.2	1	0	1	4438299	0.00%	4438299	98.4	99.94	0
ASM1490031v1	GCA_014900315.1	SAMN12325225	PRJNA555636	10/20/20	pacbio	Lineage 2.2	1	0	1	4414962	0.00%	4414962	98.4	99.94	0
ASM1490035v1	GCA_014900355.1	SAMN12325223	PRJNA555636	10/20/20	pacbio	Lineage 3	1	0	1	4431494	0.00%	4431494	98.4	99.94	0
ASM1490037v1	GCA_014900375.1	SAMN12325222	PRJNA555636	10/20/20	pacbio	Lineage 4.8	1	0	1	4419404	0.00%	4419404	98.4	99.61	0

Assembly name	GenBank assembly accession	BioSample	BioProject	Date	Sequencing_technology	Lineage	Kept_analysis	Has_illumina_raw_reads	Number_of_Contigs	Total_Length_bp	Percent_Gaps	Contigs_N50	BUSCO_Completeness	CheckM_Completeness	CheckM_Contamination
ASM1490039v1	GCA_014900395.1	SAMN12325221	PRJNA555636	10/20/20	pacbio	Lineage 2.2	1	0	1	4419030	0.00%	4419030	98.4	99.94	0
ASM1490041v1	GCA_014900415.1	SAMN12325220	PRJNA555636	10/20/20	pacbio	Lineage 2.2	1	0	1	4418657	0.00%	4418657	98.4	99.94	0
ASM1490043v1	GCA_014900435.1	SAMN12325219	PRJNA555636	10/20/20	pacbio	Lineage 2.2	1	0	1	4419694	0.00%	4419694	98.4	99.94	0
ASM1490045v1	GCA_014900455.1	SAMN12325218	PRJNA555636	10/20/20	pacbio	Lineage 2.2	1	0	1	4420004	0.00%	4420004	98.4	99.94	0
ASM1490047v1	GCA_014900475.1	SAMN12325217	PRJNA555636	10/20/20	pacbio	Lineage 2.2	1	0	1	4416797	0.00%	4416797	98.4	99.94	0
ASM1490049v1	GCA_014900495.1	SAMN12325216	PRJNA555636	10/20/20	pacbio	Lineage 2.2	1	0	1	4413235	0.00%	4413235	98.4	99.94	0
ASM1490051v1	GCA_014900515.1	SAMN12325215	PRJNA555636	10/20/20	pacbio	Lineage 2.2	1	0	1	4425422	0.00%	4425422	98.4	99.61	0
ASM1490055v1	GCA_014900555.1	SAMN12325214	PRJNA555636	10/20/20	pacbio	Lineage 3.1	1	0	1	4444842	0.00%	4444842	98.4	99.94	0
ASM1490059v1	GCA_014900595.1	SAMN12325213	PRJNA555636	10/20/20	pacbio	Lineage 2.2	1	0	1	4421377	0.00%	4421377	98.4	99.94	0
ASM1490061v1	GCA_014900615.1	SAMN12325212	PRJNA555636	10/20/20	pacbio	Lineage 2.2	1	0	1	4419973	0.00%	4419973	98.4	99.94	0

Assembly name	GenBank assembly accession	BioSample	BioProject	Date	Sequencing_technology	Lineage	Kept_analysis	Has_illumina_raw_reads	Number_of_Contigs	Total_Length_bp	Percent_Gaps	Contigs_N50	BUSCO_Completeness	CheckM_Completeness	CheckM_Contamination
ASM1490063v1	GCA_014900635.1	SAMN12325210	PRJNA555636	10/20/20	pacbio	Lineage 2.2	1	0	1	4416231	0.00%	4416231	98.4	99.94	0
ASM1490065v1	GCA_014900655.1	SAMN12325209	PRJNA555636	10/20/20	pacbio	Lineage 2.2	1	0	1	4416165	0.00%	4416165	98.4	99.28	0
ASM1490067v1	GCA_014900675.1	SAMN12325207	PRJNA555636	10/20/20	pacbio	Lineage 2.2	1	0	1	4417680	0.00%	4417680	98.4	99.83	0
ASM1490069v1	GCA_014900695.1	SAMN12325206	PRJNA555636	10/20/20	pacbio	Lineage 4	1	0	1	4413879	0.00%	4413879	98.4	99.94	0
ASM1490071v1	GCA_014900715.1	SAMN12325205	PRJNA555636	10/20/20	pacbio	Lineage 2.2	1	0	1	4413638	0.00%	4413638	98.4	99.94	0
ASM1490073v1	GCA_014900735.1	SAMN12325204	PRJNA555636	10/20/20	pacbio	Lineage 2.2	1	0	1	4419916	0.00%	4419916	98.4	99.94	0
ASM1490075v1	GCA_014900755.1	SAMN12325203	PRJNA555636	10/20/20	pacbio	Lineage 3	1	0	1	4434707	0.00%	4434707	98.4	99.94	0
ASM1490077v1	GCA_014900775.1	SAMN12325202	PRJNA555636	10/20/20	pacbio	Lineage 2.2	1	0	1	4416971	0.00%	4416971	98.4	99.94	0
ASM1490079v1	GCA_014900795.1	SAMN12325201	PRJNA555636	10/20/20	pacbio	Lineage 4.8	1	0	1	4387956	0.00%	4387956	98.4	99.94	0
ASM1490083v1	GCA_014900835.1	SAMN12325199	PRJNA555636	10/20/20	pacbio	Lineage 4.2	1	0	1	4404858	0.00%	4404858	98.4	99.94	0

Assembly name	GenBank assembly accession	BioSample	BioProject	Date	Sequencing_technology	Lineage	Kept_analysis	Has_illumina_raw_reads	Number_of_Contigs	Total_Length_bp	Percent_Gaps	Contigs_N50	BUSCO_Completeness	CheckM_Completeness	CheckM_Contamination
ASM1490085v1	GCA_014900855.1	SAMN12325198	PRJNA555636	10/20/20	pacbio	Lineage 2.2	1	0	1	4419967	0.00%	4419967	98.4	99.94	0
ASM1490089v1	GCA_014900895.1	SAMN12325197	PRJNA555636	10/20/20	pacbio	Lineage 1.2	1	0	1	4430047	0.00%	4430047	98.4	99.94	0
ASM1490096v1	GCA_014900965.1	SAMN12325196	PRJNA555636	10/20/20	pacbio	Lineage 2.2	1	0	1	4412588	0.00%	4412588	98.4	99.61	0
ASM1490103v1	GCA_014901035.1	SAMN12325195	PRJNA555636	10/20/20	pacbio	Lineage 2.2	1	0	1	4419801	0.00%	4419801	98.4	99.94	0
ASM1490105v1	GCA_014901055.1	SAMN12325194	PRJNA555636	10/20/20	pacbio	Lineage 3	1	0	1	4429476	0.00%	4429476	98.4	99.94	0.33
ASM1490107v1	GCA_014901075.1	SAMN12325193	PRJNA555636	10/20/20	pacbio	Lineage 2.2	1	0	1	4418318	0.00%	4418318	98.4	99.94	0
ASM1490109v1	GCA_014901095.1	SAMN12325192	PRJNA555636	10/20/20	pacbio	Lineage 3.1	1	0	1	4410415	0.00%	4410415	98.4	99.94	0
ASM1490111v1	GCA_014901115.1	SAMN12325191	PRJNA555636	10/20/20	pacbio	Lineage 2.2	1	0	1	4418159	0.00%	4418159	98.4	99.94	0
ASM1490113v1	GCA_014901135.1	SAMN12325190	PRJNA555636	10/20/20	pacbio	Lineage 3.1	1	0	1	4432141	0.00%	4432141	98.4	99.94	0
ASM1490115v1	GCA_014901155.1	SAMN12325189	PRJNA555636	10/20/20	pacbio	Lineage 2.2	1	0	1	4419608	0.00%	4419608	98.4	99.94	0

Assembly name	GenBank assembly accession	BioSample	BioProject	Date	Sequencing_technology	Lineage	Kept_analysis	Has_illumina_raw_reads	Number_of_Contigs	Total_Length_bp	Percent_Gaps	Contigs_N50	BUSCO_Completeness	CheckM_Completeness	CheckM_Contamination
ASM1548280v1	GCA_015482805.1	SAMN16605479	PRJNA673391	11/16/20	illumina; nanopore	M. bovis	0	1	1	4370706	0.00%	4370706	98.4	99.78	1.2
ASM1691777v1	GCA_016917775.1	SAMN17832565	PRJNA700485	2/22/21	illumina; nanopore	Lineage 2.2	1	0	1	4420561	0.00%	4420561	98.4	99.94	0
ASM1716403v1	GCA_017164035.1	SAMN18056856	PRJNA704837	3/4/21	illumina	Lineage 4.9	0	0	1	4411524	0.07%	4408665	98.4	99.94	0
ASM1716405v1	GCA_017164055.1	SAMN18056929	PRJNA704837	3/4/21	illumina	Lineage 4.9	0	0	1	4411528	0.07%	4408637	98.4	99.94	0
ASM1790103v1	GCA_017901035.1	SAMN18576181	PRJNA718842	4/12/21	illumina	Lineage 4.9	0	0	1	4432513	0.00%	4432513	98.4	99.94	0.33
ASM1790105v1	GCA_017901055.1	SAMN18576179	PRJNA718842	4/12/21	illumina	Lineage 4.9	0	0	1	4415998	0.00%	4415998	98.4	99.94	0
ASM1790107v1	GCA_017901075.1	SAMN18576180	PRJNA718842	4/12/21	illumina	Lineage 4.9	0	0	1	4415999	0.00%	4415999	98.4	99.94	0
ASM1790109v1	GCA_017901095.1	SAMN18576182	PRJNA718842	4/12/21	illumina	Lineage 4.9	0	0	1	4459449	0.00%	4459449	98.4	99.94	0
ASM1790111v1	GCA_017901115.1	SAMN18576178	PRJNA718842	4/12/21	illumina	Lineage 4.9	0	0	1	4415999	0.00%	4415999	98.4	99.94	0
ASM1967074v1	GCA_019670745.1	SAMD00322307	PRJDB11640	5/28/21	illumina; nanopore	Lineage 2.2	1	1	1	4383210	0.00%	4383210	98.4	99.94	0

Assembly name	GenBank assembly accession	BioSample	BioProject	Date	Sequencing_technology	Lineage	Kept_analysis	Has_illumina_raw_reads	Number_of_Contigs	Total_Length_bp	Percent_Gaps	Contigs_N50	BUSCO_Completeness	CheckM_Completeness	CheckM_Contamination
ASM2153515v1	GCA_021535155.1	SAMN23482338	PRJNA784324	1/20/22	nanopore	Lineage 4.9	0	0	1	4405676	0.00%	4405676	98.4	99.94	0
ASM2202399v1	GCA_022023995.1	SAMN18614372	PRJNA719670	2/7/22	illumina; pacbio	Lineage 4.4	1	1	1	4397821	0.00%	4397821	98.4	99.94	0
ASM2287012v1	GCA_022870125.1	SAMN16406479	PRJNA668291	4/11/22	nanopore	Lineage 1.1	0	0	1	4419191	0.00%	4419191	98.4	99.94	0
ASM2287014v1	GCA_022870145.1	SAMN16406478	PRJNA668291	4/11/22	nanopore	Lineage 7	0	0	1	4408017	0.00%	4408017	98.4	99.94	0
ASM2287016v1	GCA_022870165.1	SAMN16406477	PRJNA668291	4/11/22	nanopore	Lineage 6.2	0	0	1	4391569	0.00%	4391569	98.4	99.94	0
ASM2287018v1	GCA_022870185.1	SAMN16406476	PRJNA668291	4/11/22	nanopore	Lineage 6.1	0	0	1	4387551	0.00%	4387551	98.4	99.86	0
ASM2287020v1	GCA_022870205.1	SAMN16406475	PRJNA668291	4/11/22	nanopore	Lineage 6.3	0	0	1	4386429	0.00%	4386429	98.4	99.91	0
ASM2287022v1	GCA_022870225.1	SAMN16406474	PRJNA668291	4/11/22	nanopore	Lineage 5.1	0	0	1	4428969	0.00%	4428969	98.4	99.94	0
ASM2287024v1	GCA_022870245.1	SAMN16406473	PRJNA668291	4/11/22	nanopore	Lineage 5.1	0	0	1	4420832	0.00%	4420832	98.4	99.94	0
ASM2287026v1	GCA_022870265.1	SAMN16406472	PRJNA668291	4/11/22	nanopore	Lineage 5.1	0	0	1	4425207	0.00%	4425207	98.4	99.94	0

Assembly name	GenBank assembly accession	BioSample	BioProject	Date	Sequencing_technology	Lineage	Kept_analysis	Has_illumina_raw_reads	Number_of_Contigs	Total_Length_bp	Percent_Gaps	Contigs_N50	BUSCO_Completeness	CheckM_Completeness	CheckM_Contamination
ASM2287028v1	GCA_022870285.1	SAMN16406471	PRJNA668291	4/11/22	nanopore	Lineage 4.2	0	0	1	4419495	0.00%	4419495	98.4	99.94	0
ASM2287030v1	GCA_022870305.1	SAMN16406470	PRJNA668291	4/11/22	nanopore	Lineage 4.3	0	0	1	4395562	0.00%	4395562	98.4	99.94	0
ASM2287032v1	GCA_022870325.1	SAMN16406469	PRJNA668291	4/11/22	nanopore	Lineage 4.6	0	0	1	4393016	0.00%	4393016	98.4	99.94	0
ASM2287034v1	GCA_022870345.1	SAMN16406468	PRJNA668291	4/11/22	nanopore	Lineage 3	0	0	1	4431518	0.00%	4431518	98.4	99.86	0
ASM2287036v1	GCA_022870365.1	SAMN16406467	PRJNA668291	4/11/22	nanopore	Lineage 3.1	0	0	1	4438446	0.00%	4438446	98.4	99.94	0
ASM2287038v1	GCA_022870385.1	SAMN16406466	PRJNA668291	4/11/22	nanopore	Lineage 3.1	0	0	1	4422216	0.00%	4422216	98.4	99.94	0
ASM2287040v1	GCA_022870405.1	SAMN16406464	PRJNA668291	4/11/22	nanopore	Lineage 2.1	0	0	1	4399032	0.00%	4399032	98.4	99.86	0
ASM2287042v1	GCA_022870425.1	SAMN16406463	PRJNA668291	4/11/22	nanopore	Lineage 1.1	0	0	1	4420471	0.00%	4420471	98.4	99.94	0
ASM2287044v1	GCA_022870445.1	SAMN16406462	PRJNA668291	4/11/22	nanopore	Lineage 1.2	0	0	1	4410121	0.00%	4410121	98.4	99.94	0
ASM2287108v1	GCA_022871085.1	SAMN27028218	PRJNA820632	4/11/22	pacbio	Lineage 2.2	1	0	1	4413076	0.00%	4413076	98.4	99.94	0

Assembly name	GenBank assembly accession	BioSample	BioProject	Date	Sequencing_technology	Lineage	Kept_analysis	Has_illumina_raw_reads	Number_of_Contigs	Total_Length_bp	Percent_Gaps	Contigs_N50	BUSCO_Completeness	CheckM_Completeness	CheckM_Contamination
ASM2287110v1	GCA_022871105.1	SAMN27028219	PRJNA820632	4/11/22	pacbio	Lineage 2.2	1	0	1	4402590	0.00%	4402590	98.4	99.94	0
ASM2287112v1	GCA_022871125.1	SAMN27028220	PRJNA820632	4/11/22	pacbio	Lineage 2.2	1	0	1	4417237	0.00%	4417237	98.4	99.86	0
ASM2287114v1	GCA_022871145.1	SAMN27028221	PRJNA820632	4/11/22	pacbio	Lineage 2.2	1	0	1	4419329	0.00%	4419329	98.4	99.94	0
Mtb_DKC2.180803	GCA_900520315.1	SAMEA104027311	PRJEB20214	11/10/18	illumina; nanopore	Lineage 4.8	0	1	1	4409544	0.00%	4409544	98.4	99.94	0
MB3601_COMBINED annotated	GCA_902459825.2	SAMEA5803801	PRJEB33636	11/1/19	illumina; pacbio	M.bovis	0	0	1	4365068	0.00%	4365068	98.4	99.78	0
MmicOV254	GCA_904810325.1	SAMEA7336314	PRJEB40500	1/27/21	illumina; pacbio	M. microti	0	0	1	4369915	0.00%	4369915	98.4	99.94	0
MmicATCC35782	GCA_904810335.1	SAMEA7336315	PRJEB40500	1/27/21	illumina; pacbio	M. microti	0	0	1	4366549	0.00%	4366549	98.4	99.61	0
MmicMaus3	GCA_904810345.1	SAMEA7336317	PRJEB40500	1/27/21	illumina; pacbio	M. microti	0	0	1	4382575	0.00%	4382575	98.4	99.94	0
MmicMaus4	GCA_904810355.1	SAMEA7336318	PRJEB40500	1/27/21	illumina; pacbio	M. microti	0	0	1	4385022	0.00%	4385022	98.4	99.94	0
Mmic942272	GCA_904810365.1	SAMEA7336316	PRJEB40500	1/27/21	illumina; pacbio	M. microti	0	0	1	4384561	0.00%	4384561	98.4	99.61	0

Assembly name	GenBank assembly accession	BioSample	BioProject	Date	Sequencing_technology	Lineage	Kept_analysis	Has_illumina_raw_reads	Number_of_Contigs	Total_Length_bp	Percent_Gaps	Contigs_N50	BUSCO_Completeness	CheckM_Completeness	CheckM_Contamination
mada_1-44	GCA_932527315.1	SAMEA13188688	PRJEB49093	3/23/22	illumina;nanopore;pacbio	Lineage 1.1	1	1	1	4422327	0.00%	4422327	98.4	99.94	0
mada_125	GCA_932527325.1	SAMEA13188731	PRJEB49093	3/23/22	illumina;nanopore;pacbio	Lineage 2.2	1	1	1	4415120	0.00%	4415120	98.4	99.94	0
mada_116	GCA_932527465.1	SAMEA13188685	PRJEB49093	3/23/22	illumina;nanopore;pacbio	Lineage 3.1	1	1	1	4428693	0.00%	4428693	98.4	99.94	0
mada_130	GCA_932530255.1	SAMEA13188674	PRJEB49093	3/23/22	illumina;nanopore;pacbio	Lineage 4.1	1	1	1	4408536	0.00%	4408536	98.4	99.94	0
mada_132	GCA_932530315.1	SAMEA13188689	PRJEB49093	3/23/22	illumina;nanopore;pacbio	Lineage 4.8	1	1	1	4394726	0.00%	4394726	98.4	99.94	0
mada_104	GCA_932530395.1	SAMEA13188715	PRJEB49093	3/23/22	illumina;nanopore;pacbio	Lineage 1.1	1	1	1	4412157	0.00%	4412157	98.4	99.94	0
